# Phytochemical Profiling, Antioxidant, Cytotoxicity, and Anthelmintic Activity of the Bark Extracts of *Terminalia superba* Against the Murine Intestinal Nematode *Heligmosomoides polygyrus*: An Integrated In Vitro and In Silico Approach

**DOI:** 10.1155/sci5/4297066

**Published:** 2026-09-20

**Authors:** Bismarck Nganji Nsa, Yan Wu, Joan Echi Ebanga Eyong, Cédric Yamssi, Nadia A. C. Noumedem, Tientcheu N. J. Sandra, Gamago N. Guy-Armand, Mounvera A. Azizi, Ngouyamsa N. A. Sidiki, Haibo Hu, Vincent Khan Payne, Helen Kuokuo Kimbi

**Affiliations:** ^1^ Department of Biological Sciences, Faculty of Science, The University of Bamenda, P.O. Box 39, Bambili, North West Region, Cameroon, unibda.net; ^2^ Laboratory of Tropical and Emerging Infectious Diseases, Dschang, Cameroon; ^3^ National Engineering Research Center for Modernization of Traditional Chinese Medicine-Hakka Medical Resources Branch, School of Pharmacy, Gannan Medical University, Ganzhou, China, gmu.cn; ^4^ Department of Biomedical Sciences, Faculty of Health Sciences, University of Bamenda, P.O. Box 39, Bambili, Cameroon, unibda.net; ^5^ Department of Microbiology, Haematology and Immunology Faculty of Medicine and Pharmaceutical Sciences, University of Dschang, P.O. Box 96, Dschang, Cameroon, univ-dschang.org; ^6^ Department of Animal Biology, Faculty of Science, University of Dschang, P.O. Box 067, Dschang, Cameroon, univ-dschang.org; ^7^ Department of Animal Biology and Conservation (ABC), Faculty of Science, University of Buea, P.O. Box 63, Buea, Cameroon, ubuea.cm

**Keywords:** ADME, anthelmintic activity, antioxidant activity, cytotoxicity, *H. polygyrus*, *T. superba*, UHPLC, toxicity

## Abstract

**Background:**

Intestinal helminthiases are major global health concerns affecting over a billion people, causing significant morbidity, mortality, and economic losses in both humans and livestock. The growing emergence of drug‐resistant parasites has intensified the search for new, safe anthelmintic alternatives. This study evaluated the anthelmintic potential of bark extracts of *Terminalia superba* against intestinal nematode infections.

**Methods:**

Aqueous and ethanolic extracts were tested in vitro for ovicidal and larvicidal effects against *Heligmosomoides polygyrus* eggs and larval stages at concentrations ranging from 0.3125 to 5 mg/mL, in triplicate, using a high‐throughput worm microtracker. Albendazole served as positive control, and distilled water served as negative control. IC_50_ values were derived from concentration–response curves via nonlinear regression in GraphPad Prism. Antioxidant activity was assessed through DPPH, reducing power, nitric oxide, and hydrogen peroxide scavenging assays, while cytotoxicity was evaluated by hemolysis assay on human red blood cells with 1.0% Tween 20 as positive control. Phytochemical screening and UHPLC‐MS/MS fingerprinting were performed to characterize bioactive compounds. Molecular docking against succinate dehydrogenase (SDH) and *β*‐tubulin, alongside ADME predictions, were conducted using Schrödinger Maestro, with toxicity assessed via ProTox‐III.

**Results:**

Both extracts exhibited concentration‐dependent ovicidal and larvicidal activity. The aqueous extract was more effective against eggs and L1 larvae (IC_50_: 1.2 and 0.3 mg/mL), while the ethanolic extract outperformed the aqueous extract against L2 and L3 larvae (IC_50_: 0.09 and 0.8 mg/mL). The ethanolic extract demonstrated superior DPPH radical scavenging activity IC_50_: 0.75 μg/mL (95% CI: 0.14–1.62), compared to the aqueous extract, 6.26 μg/mL (4.07–9.50), and vitamin C, 1.91 μg/mL (1.38–2.55). Both extracts showed minimal cytotoxicity, with hemolysis of 7.6% and 2% at 1000 μg/mL, respectively. Phytochemical analysis revealed a rich profile of tannins, flavonoids, and phenolic compounds, with UHPLC‐MS/MS confidently identifying 82 of over 300 detected features. Top docking candidates against *β*‐tubulin included kaempferol (−8.033), brevifolin‐carboxylic acid (−7.985), luteolin (−7.954), scutellarein (−7.373), and (−)‐epicatechin (−6.980), all surpassing albendazole (−3.728). Against SDH, leading compounds were termilignan (−6.767), cyanidin (−6.659), hesperetin (−6.314), combretastatin A‐1 (−6.274), and luteolin (−6.166), outperforming albendazole (−5.541). ADME profiling of these leading compounds indicated favorable oral absorption and bioavailability. ProTox‐III classified all top compounds at low toxicity levels (class ≥ 4).

**Conclusion:**

This study offers preliminary scientific support for the traditional use of *T. superba* bark extracts in managing intestinal helminthiases, warranting further in vivo validation.

## 1. Introduction

Among the most common infections worldwide are parasitic infections, responsible for an enormous burden of disease, economic loss, and preventable deaths. Parasites from different classes can live in the human digestive tract and cause diseases in the gut (intestinal parasitoses). Intestinal helminthiases have been a major cause of health problems in humans and livestock since ancient times, inflicting a serious economic loss among populations. *Ascaris lumbricoides*, *Trichuris trichiura*, *Enterobius vermicularis*, hookworms (*Ancylostoma duodenale* and *Necator americanus*), and *Strongyloides stercoralis* are human gastrointestinal nematodes of medical significance that cause morbidity and mortality in humans [1]. Tropical and subtropical populations are heavily affected, particularly affecting children in Sub‐Saharan Africa, Latin America, China, and East Asia accounting for the bulk of infections [2].

Soil‐transmitted helminth (STH) infections remain a major problem for public health around the world. They affect more than 1.5 billion people, representing about 24% of the world’s population [3]. Recent reports from the World Health Organization indicate that more than 800 million children need preventive chemotherapy, especially in countries with low or middle incomes [3]. A large part of this burden falls on Sub‐Saharan Africa, where transmission is closely linked to poor sanitation and limited access to healthcare. Even though whole populations could be geographically at risk, children are observed to disproportionably carry the highest burden of infection [4]. In Cameroon, STH infections remain endemic, particularly within vulnerable populations, underscoring the necessity for ongoing research into effective and accessible control strategies.

The rise in expenses, the unavailability of current chemotherapies, ongoing reinfection, the development of drug‐resistant parasites, and the negative consequences linked to widespread medication use have become major drawbacks to the success of anthelmintic chemotherapy [5, 6]. Additionally, the lack of coverage for other infectious agents like *Strongyloides* further limits the effectiveness of current control strategies [7]. These threats have prompted the pursuit of discovering and developing novel, innovative, sustainable, effective, safe, alternative, and complementary therapeutic alternatives, primarily derived from natural sources [8, 9].

Intestinal nematode parasites cause large lesions of the epithelia [10]. Phagocytes intervene following the presence of helminths or the lesions they cause by inducing the release of free radicals, which can help kill the parasites. The excessive production of free radicals including the reactive oxygen species (ROS) and reactive nitrogen species (RNS) can distort this equilibrium, leading to oxidative stress [11, 12], which, if left untreated, severely destroys proteins, lipids, and DNA, resulting in cellular malfunction [13]. Antioxidant plant extracts may reduce oxidative damage and inflammation in the host during helminth infection [14].

As sources of anthelmintic compounds and possible antioxidants, medicinal plants have considerable potentials. Some plant extracts contain bioactive compounds such as tannins, flavonoids, and alkaloids, which demonstrate both antioxidant and anthelmintic properties in independent mechanisms [15, 16]. Plants have long been used in traditional medicine for various illnesses [17], but their safety and efficacy are not assured, as some may be toxic and require thorough scientific validation. Approximately 80% of Africans mostly rely on traditional treatments for their medical requirements [18, 19]. These treatments being part of folkloric practices are seen to be safe and effective, more readily available, and less expensive than conventional medications [9, 20].

Among the rich biodiversity of plants in Africa is *Terminalia superba*, a plant (tree) in the genus *Terminalia*, which grows in the tropical regions of the world and is widespread in West and Central Africa [21]. *T. superba* commonly known as *“Akom”* in Cameroon is locally used in the treatment of various ailments, including fever, leprosy, piles, liver problems, cough, asthma, and stomach pain [22]. Its numerous pharmacological properties, among which hypoglycemic [23], antibacterial [24, 25], and antifungal activities [25, 26], are well established. According to Dongmo et al. [27], traditional healers use *T. superba* and other plants in the family (Combretaceae) to treat a variety of ailments, including heart disease, worms, gastric ulcers, diarrhea, dysmenorrhea, cough, colds, conjunctivitis, and gastrointestinal disorders. Other species of the genus such as *T. macroptera, T. glaucescens*, and *T*. *catappa* have been shown to exhibit anthelmintic activity [28–30], and these effects have been linked to their bioactive secondary metabolites [15]. The qualitative phytochemical analysis of the extracts of *T. superba* leaves and roots revealed their richness in alkaloids, phenols, tannins, flavonoids, saponins, coumarins, quinones, proteins, and amino acids [31, 32].

Given its rich phytochemical composition and demonstrated biological activities, *T. superba* is a strong candidate for anthelmintic evaluation. Investigating its potential could not only validate its traditional uses but also contribute to the discovery of novel, plant‐based anthelmintic agents capable of addressing the growing challenge of drug resistance. Despite its traditional use against worms, no systematic phytochemical profiling combined with in vitro and in silico evaluation has been conducted.

This study evaluated the phytochemical profile, antioxidant, cytotoxic, and anthelmintic activities of *T. superba* bark extracts, alongside their in silico molecular docking interactions and pharmacokinetic properties using *Heligmosomoides polygyrus* as a model intestinal nematode. Because of its reproducible life cycle, ease of maintenance, and relevance to host‐parasite interactions, *H. polygyrus* is a well‐established experimental model for gastrointestinal nematode infections that is frequently used in laboratory studies [33]. It also shares important biological and molecular characteristics with human STHs [34].

## 2. Materials and Methods

### 2.1. Collection and Identification of Plant Parts

The stem barks of the plant (*T. superba* Engl. and Diels) were collected from Magba subdivision, Noun Division, West Region of Cameroon. Magba is found in the Bamoun plateau, some 735 m above sea level, occupying latitude 5°57′N and longitude 11°13′E. The collection was done in the rainy season, precisely in the month of September 2024. The samples of the specimens collected were transported to Yaoundé, where they were carefully examined and confirmed as *T. superba* at the National Herbarium of Cameroon in Yaoundé. The plant species was identified and registered under Voucher No. 1875SRFK.

### 2.2. Preparation of Aqueous and Ethanolic Extracts

The plant material was air‐dried under shade in a well‐ventilated environment at ambient temperature (25–30°C) for 7 days. During this drying, samples were spread out periodically to ensure uniform airflow and were kept in a clean, dry indoor environment to prevent moisture uptake and contamination. It was later ground to powder form and stored at room temperature. The extracts were prepared using the procedure outlined by Wabo‐Poné et al. [35]. Whatman paper No. 1 (pore size ∼11 μm) was used to filter the ethanolic extract after 100 g of the powder was macerated in 1 L of 95% ethanol for three days (72 h) with vigorous shaking every day. For the aqueous extracts, 100 g of the powder was first filtered through a screen (tea strainer), followed by Whatman paper No. 1 (pore size ∼11 μm), after being macerated in 1 L of heated distilled water at 100°C for three hours. The filtrates were dried at 40°C in an oven (BIOBASE), after which the dried extracts were collected and stored at 4°C for subsequent use. The ethanolic extract was dried to constant weight to ensure complete removal of residual ethanol. The percentage yield was calculated using the following formula:

(1) % yield=mass of extract obtained initial mass of plant material×100.

### 2.3. The Parasite Culture

Fifteen milliliters (15 mL) of distilled water containing 7500 L3 larvae of *H. polygyrus* in suspension were obtained from The Biological Resource Center, the American Type Culture Collection (ATCC) in the United States. The parasites were used to infect 8–10 weeks old, 20–30 g mice of both sexes orally by gavage.

Eggs were collected using the flotation technique as elaborated by Cédric et al. [36]. The collected eggs were washed 3 times in distilled water by centrifugation at 1500 rpm for 5 min each. The salt‐free eggs were then cultured to obtain the different life cycle stages (embryonated eggs, L1 and L2 larvae) of the parasite. The aforementioned life cycle stages were obtained by adding about 12 mL of the fresh egg suspension into petri dishes and incubating the cultures at room temperature (25°C ± 2°C) for 1 day (24 h), 2 days (48 h), and 4 days (96 h) for embryonated eggs, and L1 and L2 larval stages of *H. polygyrus*, respectively [36]. The L3 larval stage was obtained from the culture of fresh feces by the method described by Ref. [37]. After washing charcoal under running water for at least half an hour, it was allowed to air dry. Then, the charcoal was ground into a powder and combined with freshly ground feces from infected mice in a 1:1 ratio. A tiny amount of water was added just enough to create a paste. The L3 larvae were collected after the mixture of excrement, and charcoal was spread on a thin layer of moist filter paper in a Petri dish and incubated for 7 days in a humid box in the dark.

### 2.4. In Vitro Anthelmintic Activity

The dried extracts were reconstituted in distilled water to prepare stock solutions prior to the assays. Distilled water was used as the negative control, while albendazole served as the positive control.

The ovicidal and larvicidal properties of the aqueous and ethanolic extracts were assessed using the procedure outlined by Cedric et al. [36].

About 50 embryonated eggs and larvae for the larval stages (L1, L2, and L3) in 100 μL of distilled water were put in contact with 100 μL of extracts in triplicate at different concentrations (5, 2.5, 1.25, 0.625, and 0.3125 mg/mL) in a 96‐well round bottomed microtiter plate. Albendazole prepared at the same varied concentrations (5, 2.5, 1.25, 0.625, and 0.3125 mg/mL) was used as positive control, and distilled water was used as negative control. The different setups were incubated at 25°C for 24 h in the worm microtracker (Phylumtech, Argentina) device. Motility of the worms was measured every 30 min. Although motility was monitored during the incubation period, the determination of anthelmintic activity was based on analysis of the 24‐h endpoint data. The percentage inhibition of egg hatching and percentage inhibition of larval motility of worms was calculated using the following formula [38]:

(2) % inhibition=mobility activity of negative control−mobility activity of the test samplemobility activity of negative control×100.

### 2.5. In Vitro Antioxidant Activity of *T. superba* Extracts

#### 2.5.1. 2,2‐Diphenyl‐1‐Picrylhydrazyl (DPPH) Radical Scavenging Activity (RSA)

The method developed by Popovici et al. [39] was used to evaluate the extracts’ capacity to scavenge DPPH free radicals. The basis of this test is the reduction of DPPH^∗^ in methanol in the presence of an electron or hydrogen donor, which results in the creation of the nonradical form DPPH‐H [39]. The transformation results in a color change from violet to yellow, and absorbance was measured at 517 nm using a spectrophotometer (BIOBASE BK‐D560). In the spectrophotometer wells, 0.5 mL of plant extracts or vitamin C, at the different final concentrations (1, 3, 10, 30, 100, and 300 μg/mL), was mixed with 0.5 mL of methanol and 0.5 mL DPPH (0.063 mg/mL). The mixture was incubated in the dark at room temperature (25°C) for 20 min. After incubation, absorbance was measured at 517 nm. Methanol was used as the blank, while 0.5 mL of DPPH solution and 1 mL of methanol served as the negative control. Ascorbic acid was used as a positive control. All experiments were performed in triplicate. The RSA of DPPH was calculated as percentage inhibition using the following formula:

(3) RSA of DPPH=absorbance of control−absorbance of test sample absorbance of control ×100.

#### 2.5.2. Reducing Power Assay

This test was carried out using the procedure outlined by Bokhari et al. [40], which measures the antioxidant capability of a substance by evaluating its ability to convert ferric ions (Fe^3+^) to ferrous ions (Fe^2+^). Briefly, a spectrophotometer (BIOBASE BK‐D560) was used to measure absorbance at 700 nm after 200 μL of each plant extract or vitamin C at concentrations (1, 3, 10, 30, 100, and 300 μg/mL) was combined with 0.5 mL of phosphate buffer (200 mM, pH 6.6) and 0.5 mL of potassium ferricyanide [K_3_Fe(CN)_6_] solution (30 mM), and then, the mixture was incubated at 37°C for 10 min. A blank containing all reagents except the extract was prepared and treated under identical conditions. Ascorbic acid (vitamin C) served as a positive control. All treatments were performed in five technical replicates.

#### 2.5.3. Nitric Oxide (NO) Radical Inhibition Assay

The Griess Ilosvay reaction was used to estimate the inhibition of NO radicals as described by Ashokkumar et al. [41] with slight modification.We used sodium nitroprusside (Na_2_[Fe(CN)_5_NO]; 10 mM) in phosphate‐buffered saline (pH 7.4) as a source of NO. The NO generated in vitro reacts with oxygen to produce nitrite ions, which the Griess reaction measures. A sock solution of about 2.8 mg/mL was obtained by dissolving 10 mg of extract or vitamin C in 3.53 mL of phosphate‐buffered saline (pH = 7.4; 10 mH; pKa 6.9). A volume of 1520 μL of sodium nitroprusside (10 mM) was added to each test tube containing 180 μL of extract or vitamin C (at the various final concentrations: 1, 3, 30, 100, and 300 μg/mL). For 150 min, the mixture was incubated at room temperature (25°C) under light to facilitate NO generation. Next, 500 μL of the mixture was put into wells, followed by the addition of an equivalent volume of 1% sulfanilamide, then homogenized, and incubated for 5 minutes at room temperature in the dark. Finally, 500 μL of naphthyl ethylene diamine (NED, 0.1%) was added into the mixture and the mixture was then incubated in the dark for 5 minutes.Phosphate‐buffered saline served as the blank, and sodium nitroprusside without extract was the negative control. The optical density (OD) of the resultant chromophore was measured within 30 min at 530 nm (530–546 nm). The percentage of RSA was calculated using the following formula [42]:

(4) RSA of NO=ODcontrol−ODsampleODcontrol ×100.

#### 2.5.4. Hydrogen Peroxide (H_2_O_2_) Scavenging

The plant extracts’ capacity to scavenge hydrogen peroxide was assessed using the technique developed by Ruch et al. [43]. A solution of hydrogen peroxide (40 mM) was prepared in phosphate buffer (pH 7.4, 50 mM). Briefly, a volume 0.4 mL of extracts or ascorbic acid (positive control) at several concentrations (1, 3, 10, 30, 100, 300 μg/mL) were added to 0.6 mL of H_2_O_2_ solution and the mixture was incubated at room temperature (25 ± 2°C) for 10 min. The absorbance was measured at 230 nm using a blank solution of phosphate buffer that did not contain hydrogen peroxide. A control solution without extract but containing hydrogen peroxide was also formulated. All measurements were done in triplicate. Scavenging percentage was assessed as

(5) H2O2 % scavenging=absorbance of control−absorbance of test sample absorbance of control ×100.

### 2.6. Cytotoxicity Test (Hemolysis Assay)

The hemolysis test was assessed according to the method described by Ref. [44], with slight modifications. Fresh venous blood collected into EDTA tubes from consented donors was centrifuged at 1500 rpm (226 × g) for 5 min and washed three times with 0.9% NaCl solution, and a 4% hematocrit was prepared. A 1.0% Tween 20 stock solution served as positive control, while erythrocyte suspension in 4% hematocrit served as the negative control. Briefly 500 μL of 4% erythrocyte suspension was mixed with 500 μL of extract at concentrations ranging from 31.25 to 1000 μg/mL (in quadruplicates) and with 1.0% Tween 20. The mixtures were incubated at 37°C for 3 h and then centrifuged at 2500 rpm (629 × g) for 3 min, after which 100 μL of supernatant was transferred to a 96‐well microplates, and absorbance was read at 540 nm using a BIOBASE microplate reader. PBS‐treated erythrocytes were used as the negative control, while PBS alone was used as the blank. The percentage of hemolysis was calculated using the following formula:

(6) percentage of hemolysis %=OD extract−OD negative control OD positive control−OD negative control ×100.

### 2.7. Phytochemical Screening of *T. superba* Extracts

#### 2.7.1. Qualitative Phytochemical Screening

Aqueous and ethanol extracts of the stem bark of *T. superba* were screened for the presence of secondary metabolites such as alkaloids, flavonoids, phenols, triterpenoids, quinones, and tannins, using the Dragendorff test, conc. H_2_SO_4_ test, potassium dichromate test, Salkowski’s test, conc. HCl test, and the 10% NaOH test, respectively, using the methods described by Shaikh and Patil [45].

#### 2.7.2. Quantitative Phytochemical Screening

##### 2.7.2.1. Determination of Total Phenolic Content

The Folin–Ciocalteu reagent was used to determine the level of total phenols in each extract [46]. Briefly, 0.5 mL of extract (100 μg/mL or 0.1 mg/mL) in triplicate was mixed with 1.25 mL of 75% (w/v) saturated sodium carbonate solution. The mixture was homogenized and incubated at 40°C for 30 min. The optical density was read with a spectrophotometer at 765 nm. Gallic acid was used as a positive control. The total phenolic compound contents were expressed as milligrams of gallic acid equivalent per gram of extract. The amount of polyphenols in the extracts was calculated using the equation for the calibration line.

##### 2.7.2.2. Determination of Total Flavonoid Content

The total flavonoid content was measured using the method elaborated by Zhishen et al. [47], with slight modifications. To test tubes in triplicate containing 0.5 mL of each extract of concentrations 500 μg/mL (0.5 mg/mL), 150 μL of sodium nitrite (5%) and 1.5 mL of distilled water were added. After 5 min of incubation at room temperature (25°C ± 2°C), 150 μL of 10% aluminum chloride (prepared in methanol) was added, and the reaction was allowed for an additional 6 min. Then, the reaction medium was homogenized after adding 500 μL of 4% NaOH solution. Distilled water was then used to increase the reaction volume to 5 mL. A spectrophotometer set to 510 nm was used 10 minutes later to measure the color’s intensity. The amount of flavonoids contained in the extracts was calculated using the equation of the calibration.

### 2.8. UHPLC‐MS/MS Phytochemical Profiling

For the sample processing, approximately 1.0 g of *T. superba* stem bark sample was accurately weighed and mixed with 10 mL of mass spectrometry‐grade methanol. Ultrasonic‐assisted extraction was subsequently performed (3 × 30 min, with a 4–6‐h interval between each cycle). Following extraction, the supernatants were collected, combined, and filtered through a 0.22‐μm micropore membrane. The filtrate was then diluted 20‐fold prior to analysis. All procedures were conducted with three parallel replicates, and corresponding blank control groups were established throughout the experiment. Before UHPLC‐MS/MS analysis, the extracts were kept in airtight containers at −20°C and out of the light. The analyses took place within 10 days of extraction.

UHPLC‐MS/MS analysis was conducted using an Agilent 1290 Infinity II series liquid chromatography system coupled with an Agilent G6560C Q‐TOF mass spectrometer. The LC system was equipped with a binary pump (G7120A), an autosampler (G7129B), and a diode array detector (G7117A). Chromatographic separation was performed on an Agilent ZORBAX Eclipse Plus C18 column (2.1 × 50 mm, 1.8 μm; RRHD) maintained at 40°C. The mobile phase consisted of water (A) and acetonitrile (B) at a flow rate of 0.350 mL/min. The gradient elution program was set as follows: 0–2 min, 5% B; 2–26 min, 5%–100% B; 26–29 min, 100% B; 29.01–30 min, and 100%–5% B, followed by re‐equilibration. The injection volume was 1.0 μL. DAD signals were acquired at wavelengths of 254 nm, 210 nm, and 365 nm. To ensure analytical consistency, an internal standard was added to each sample before analysis. Peak areas facilitated semiquantitative analysis for comparing compound abundance, while external calibration curves were created for major compounds using authentic standards to allow approximate quantification. Calibration curves were established over suitable concentration ranges, confirming linearity with *R* ^2^ ≥ 0.99. Quantification was performed based on corresponding calibration curves.

### 2.9. Molecular Docking

**FIGURE 1:**
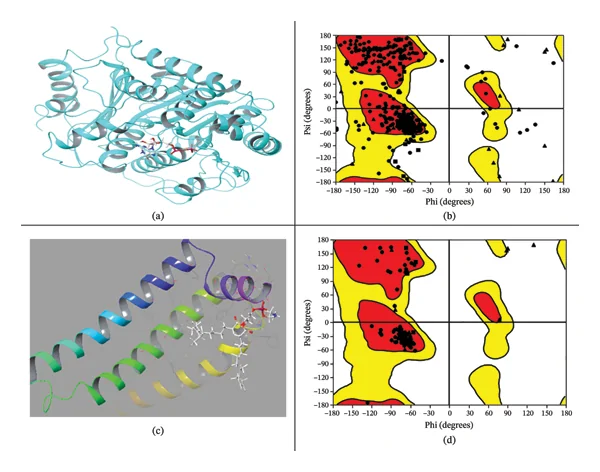
3D structures and Ramachandran plot validation of prepared *β*‐tubulin (PDB ID: 1SA0) and succinate dehydrogenase (PDB ID: 4YSX) receptors. For the *β*‐tubulin: (a) optimized *β*‐tubulin receptor, and (b) Ramachandran plot of *β*‐tubulin receptor protein. For the SDH protein: (c) optimized SDH model receptor and (d) Ramachandran plot of SDH receptor protein.

According to data from multiple publications, the anthelmintic receptor proteins; the *β*‐tubulin protein [48, 49] and succinate dehydrogenase (SDH) [50–52] were selected as the targets for this investigation. These protein targets were downloaded from the Protein Data Bank, namely, *β*‐tubulin‐colchicine protein (PDB ID: 1SA0) and SDH (PDB ID: 4YSX). Both proteins were generated and prepared using the Protein Preparation Wizard of Schrödinger Maestro (Version 12.5) as seen in Figure 1. Biological units were added, bond orders were assigned, disulfide bonds were made, all the crystallographic water molecules that did not have 3H bonds were removed, metal‐binding states for heteroatoms were produced, and missing hydrogens were added using the Protein Preparation Wizard. Protein protonation states were determined at pH 7.0 ± 2.0 using PROPKA [53], and the crystal structure’s energy was minimized using the OPLS4 force fields. Using the SiteMap module of Schrödinger Maestro, we identified the active binding pockets of *β*‐tubulin and SDH. The top binding site, selected according to SiteScore and Dscore values, was used to create the receptor grid. The *β*‐tubulin and SDH chain grid boxes were generated using the Glide receptor grid generation program from Maestro, since its Sitemap program can anticipate the receptor’s active location. For *β*‐tubulin, two grid box sizes were set: a smaller box (15 × 15 × 15 Å) centered at (X: 93.05, Y: 73.26, Z: 0.15) to allow focused docking at the predicted active site, and a larger box (20 × 20 × 20 Å) to allow semiblind exploration of the protein surface. For SDH, grid boxes of 10 × 10 × 10 Å and 20 × 20 × 20 Å were also centered at (X: 113.92, Y: 8.28, Z: 60.79).

Eighty‐two ligands identified from UHPLC‐MS profiling of *T. superba* bark extract were retrieved from PubChem and prepared using the LigPrep module [54]. The 3D conformation of the ligands were obtained from the PubChem online portal. Protonation states and tautomeric forms were assigned at pH 7.0 ± 2.0 using Epik [55], and stereoisomers were either retained where defined or fully enumerated where undefined.

The Glide module of the Schrödinger Maestro software was used to conduct the docking studies [54]. For every ligand, up to 10 docking poses were generated and the one with the lowest docking score and the best interaction profile was chosen. The software’s scoring function was used to classify and rank a number of potential adduct structures produced by molecular docking [56]. It uses the binding properties of the ligand and target to predict the three‐dimensional structure of any complex. Docking was conducted in both standard precision (SP) and extra precision (XP) modes. Compounds were first screened using SP docking and then by XP docking of the top‐ranked ligands to refine binding poses and improve scoring and accuracy. The ligand–receptor complexes were ranked by GlideScore (kcal/mol). Using Maestro (Schrödinger), protein–ligand interactions such as hydrogen bonding, hydrophobic interactions, and π–π stacking interactions were analyzed and visualized.

### 2.10. In Silico Absorption, Distribution, Metabolism, and Excretion (ADME)/Pharmacokinetics Predictions

The Schrödinger Maestro QikProp module (Version 12.5) was used to predict the ADME properties of the lead compounds that presented the best docking scores with the target proteins, *β*‐tubulin, and SDH. The physicochemical and pharmacokinetic properties of these prominent inhibitors were assessed in order to forecast their drug likeness. The simulation assesses outliers using Lipinski’s rule of five and produces ranges based on the characteristics of 95% of known medications [57].

The ligands were subjected to the Lipinski rule of five, and in order to eliminate hits with more than one violations, numerous physiological parameters were also assessed as stated by Dash et al. [58]. Table 1 shows the parameters evaluated and the cutoffs used to define acceptable pharmacokinetic properties.

**TABLE 1:** QikProp ADME property ranges.

Property	Descriptor	Normal range (95% of drugs)	Interpretation
QPlogPo/w	Octanol/water partition coefficient	−2.0 ⟶ +6.5	Balance between lipophilicity and hydrophilicity
QPlogS	Predicted aqueous solubility (mol/L)	−6.5 ⟶ 0.5	Higher = more soluble
QPPCaco	Caco‐2 permeability (nm/s)	< 25 = poor; 25–500 = moderate; > 500 = great	Proxy for intestinal absorption
QPlogBB	Brain/blood partition coefficient	−3.0 ⟶ +1.2	> 0 ⟶ CNS penetration likely
%Human oral absorption	Predicted absorption	< 25% = low; 25%–80% = medium; > 80% = high	Bioavailability
PSA	Polar surface area (Å^2^)	< 140 (general drugs); < 90 (CNS drugs)	Lower PSA favors permeability
#Stars	# of descriptors outside 95% range	0–5 = drug‐like	More = less drug‐like
Rule of five violations	Lipinski’s filter	≤ 1 acceptable	Higher ⟶ poor oral bioavailability
QPlogHERG	Predicted IC50 for HERG K+ channel (cardiotoxicity)	Concerns if < −5	Risk of arrhythmia
QPlogKhsa	Binding to human serum albumin	−1.5 ⟶ +1.5	Outside range = unusual PK

### 2.11. Toxicity Predictions of the Leading Ligands

The ProTox‐III web tool was used to investigate toxicity prediction. It is an important tool for predicting the toxicity of small compounds. Following the docking analysis, toxicological investigations were conducted on the ligand with the best docking results [59].

The endpoints predicted were acute toxicity (LD_50_), hepatotoxicity, nephrotoxicity, mutagenicity, carcinogenicity, and immunotoxicity. The predictions were made based on administering the drug by the oral route to rodents, with LD_50_ values given in mg/kg body weight. The globally harmonized system was used to classify compounds into toxicity classes (I–VI), with Classes IV–VI showing relatively low toxicity. These predictions were used to help choose the best lead compounds by prioritizing molecules with higher LD_50_ values and good toxicity profiles.

### 2.12. Ethical Considerations

The “Principles for the care of Laboratory Animals” (NIH Publication No. 85‐23, amended 1985) were scrupulously followed by all authors. Particular national legislation pertaining to the topic was also taken into account. Ethical approval for this study was obtained from the Institutional Ethics Committee for Research on Human Health Review Board of the University of Douala, Cameroon, with approval no. N°4112/IEC‐UDo/01/2024/T.

All animal experiments adhered to institutional guidelines for laboratory animal care, with animals kept under controlled conditions (25 ± 2°C; 12‐h light/dark cycle, with free access to food and water) and measures taken to minimize pain and distress. Human blood samples were collected from healthy volunteers with ethical approval and informed consent, in line with established ethical guidelines.

### 2.13. Statistical Analyses

All experiments were performed in triplicate (*n* = 3). The percentage inhibition was calculated using Microsoft Excel based on the activity values. Concentration (dose) response curves were created, and 50% inhibitory concentration (IC_50_) values were determined using nonlinear regression analysis in GraphPad Prism Version 8 software. The IC_50_ values are reported with 95% confidence intervals (CIs). The independent samples *t* tests were used to compare the quantitative phytochemical contents of the aqueous and ethanolic extracts, with statistical significance set at *p* < 0.05. The Schrödinger software listed and ranked several structures for adducts created by molecular docking based on their docking scores. The Schrödinger Maestro 12.5 QikProp tool was used to calculate the ADME properties of compounds.

## 3. Results

### 3.1. Percentage Yield of the *T. superba* Bark Extracts

Extraction of 100 g of dried bark powder yielded 11.12 g (11.12% w/w) of ethanolic extract and 20.65 g (20.65% w/w) of aqueous extract.

### 3.2. Anthelmintic Activity of *T. superba* Extracts

The inhibitory effects of *T. superba* extracts on *H. polygyrus* egg hatching and larval development were investigated across a range of concentrations (5, 2.5, 1.25, 0.625, and 0.3125 mg/mL). A concentration‐dependent effect was observed across all treatments.

**FIGURE 2:**
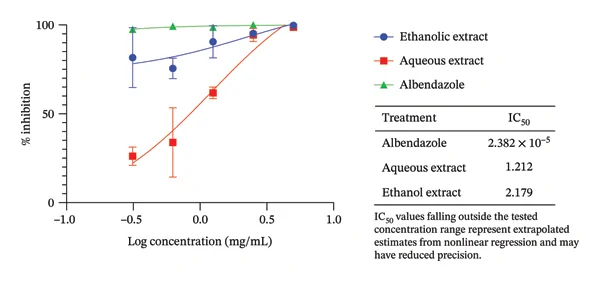
Ovicidal activity of extracts of *T. superba*.

The ovicidal activity of the aqueous and ethanolic extracts of *T. superba* on embryonated eggs of *H. polygyrus* is illustrated in Figure 2. The percentage (%) inhibition of hatching of the embryonated eggs is plotted against the log of concentrations. This figure shows that albendazole (positive control) showed the highest percentage inhibition of hatching with an IC_50_ value of 2.4 × 10^−5 ^mg/mL; meanwhile, distilled water (negative control) showed a 100% egg hatching showing no inhibitory effects on worm hatching. The extracts of *T. superba* stem bark showed a concentration‐dependent inhibition of hatching, with the highest concentration (5 mg/mL) showing a 100% hatching inhibition. The aqueous extract was more active than the ethanolic extract with IC_50_ values of 1.2 mg/mL against 2.1 mg/mL, respectively. In comparison with the tested samples, albendazole showed a much lower IC_50_ value, indicating improved anthelmintic efficacy.

**FIGURE 3:**
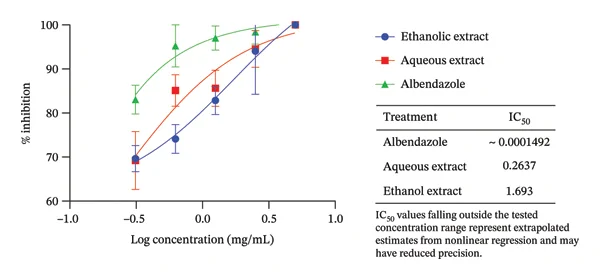
Larvicidal activity of extracts of *T. superba* against the L1 larval stage.

The L1 larvicidal activity is illustrated by Figure 3. The extract activity was concentration dependent. The aqueous and ethanolic extracts showed IC_50_ values of 0.3 and 1.7 mg/mL, respectively, indicating that the aqueous extract was more active than the ethanolic extract. Albendazole demonstrated a markedly reduced IC_50_ value (0.0001 mg/mL), signifying enhanced anthelmintic efficacy relative to the examined samples.

**FIGURE 4:**
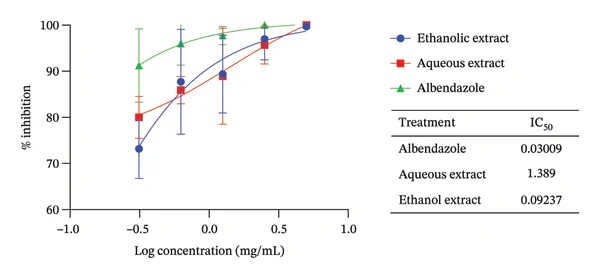
Larvicidal activity of extracts of *T. superba* against the L2 larval stage.

Figure 4 shows the L2 larvicidal activity of *T. superba* extracts. Distilled water (negative control) showed a 100% larval development, indicating no larvicidal effect on the development of the parasites, while albendazole (positive control) showed the highest larvicidal activity. The larvicidal activity of both extracts was concentration dependent, with the ethanolic extract being more active than the aqueous extract with IC_50_ values of 0.09 mg/mL for the ethanolic extract and 1.4 mg/mL for the aqueous extract, respectively. These larvicidal activities were lower than that of albendazole with an IC_50_ of 0.03 mg/mL, indicating an improved efficacy of albendazole.

**FIGURE 5:**
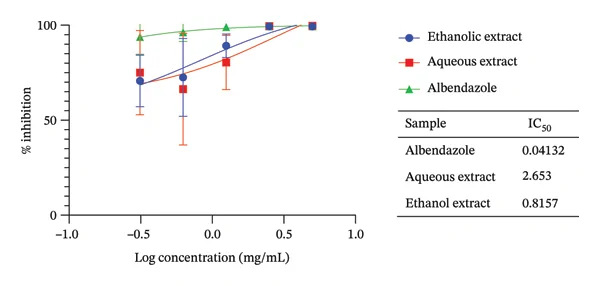
Larvicidal activity of extracts of *T. superba* against the L3 larval stage.

Figure 5 shows the larvicidal impact of the ethanolic and aqueous extracts of *T. superba* on *H*. *polygyrus* L3 larvae, with IC_50_ values of 0.8 and 2.6 mg/mL, respectively. This shows that the ethanolic extract had a greater larvicidal effect on the L3 larvae than the aqueous extract. Albendazole (positive control) showed the highest larvicidal activity (IC_50_ of 0.04 mg/mL), while distilled water (negative control) showed no effect on the larvae development.

### 3.3. NO Radical Inhibition of *T*. *superba* Extracts

**FIGURE 6:**
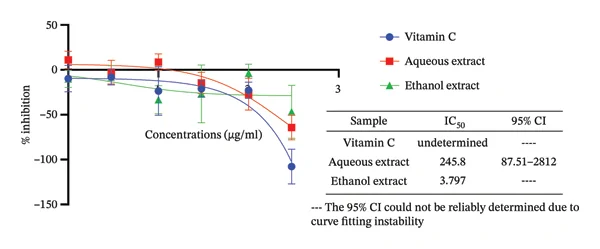
Nitric oxide (NO) radical inhibition activities of *T. superba* extracts.

Figure 6 shows the results obtained from the NO radical inhibition activities of *T*. *superba* extracts. Aqueous extract demonstrated a NO scavenging activity with an IC_50_ of 245.8 μg/mL (95% CI: 87.51–2812 μg/mL), while the ethanolic extract showed a notably lower IC_50_ of 3.8 μg/mL, indicating stronger reducing power, although its 95% CI could not be reliably determined due to instability in curve fitting. The IC_50_ of the reference standard, Vitamin C, could not be determined within the tested concentration range. These results suggest that the ethanolic extract possesses considerably greater reducing power compared to the aqueous extract.

### 3.4. DPPH RSA of *T. superba* Extracts

**FIGURE 7:**
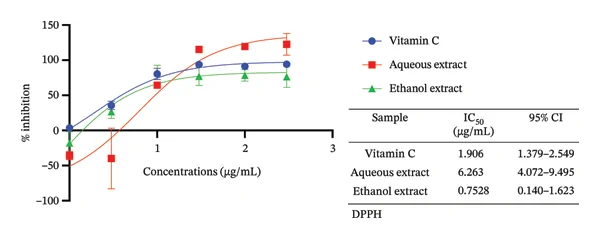
DPPH radical scavenging activities of *T. superba* extracts.

Figure 7 displays *T. superba* extracts’ capacity to scavenge DPPH radicals. This figure demonstrates that both the ethanolic and aqueous extracts strongly inhibited DPPH’s antiradical activity, with the ethanolic extract having the highest scavenging capacity, indicating a moderately higher activity than vitamin C. Aqueous and ethanolic extracts had IC_50_ values of 6.263 μg/mL (95% CI: 4.072–9.495 μg/mL) and 0.7528 μg/mL (95% CI: 0.140–1.623 μg/mL), respectively, while vitamin C (positive control) had an IC_50_ value of 1.906 μg/mL (95% CI: 1.379–2.549 μg/mL). These results indicate that the ethanolic extract exhibited the strongest DPPH RSA, followed by vitamin C.

### 3.5. Reducing Power of *T. superba* Extracts

**FIGURE 8:**
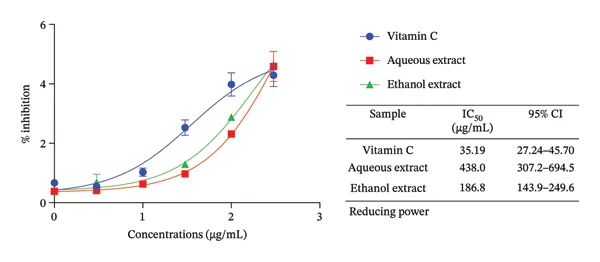
Reducing power of *T. superba* extracts.

Figure 8 displays the reducing power capacity of the ethanol and aqueous extracts of *T*. *superba* together with vitamin C. Vitamin C exhibited the highest reducing power (IC_50_: 35.19 μg/mL; 95% CI: 27.24–45.70 μg/mL), followed by the ethanolic extract (IC_50_: 186.8 μg/mL; 95% CI: 143.9–249.6 μg/mL) and the aqueous extract (IC_50_: 438.0 μg/mL; 95% CI: 307.2–694.5 μg/mL). The extracts showed lower reducing antioxidant power compared to vitamin C; meanwhile, the ethanolic extract showed a better potency compared to the aqueous extract.

### 3.6. Hydrogen Peroxide (H_2_O_2_) Scavenging Activity of *T. superba* Extracts

**FIGURE 9:**
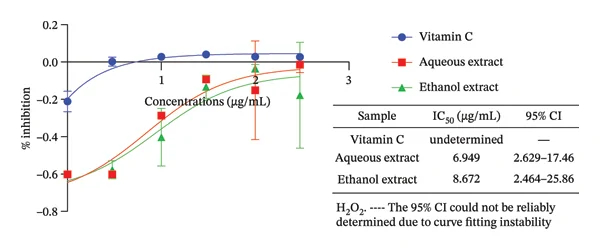
Hydrogen peroxide (H_2_O_2_) scavenging activity of *T. superba* extracts.

The H_2_O_2_ radical scavenging abilities of the aqueous and ethanolic extracts of *T. superba* are shown in Figure 9. Both extracts demonstrated measurable H_2_O_2_ scavenging activity, with the aqueous extract exhibiting a greater potency with IC_50_ value 6.949 μg/mL (95% CI: 2.629–17.46 μg/mL) compared to the ethanolic extract with an IC_50_ value of 8.672 μg/mL (95% CI: 2.464–25.86 μg/mL). The IC_50_ of the reference standard vitamin C could not be determined within the tested concentration range.

### 3.7. Cytotoxic Activities of the Aqueous and Ethanol Extracts of *T. superba*

**FIGURE 10:**
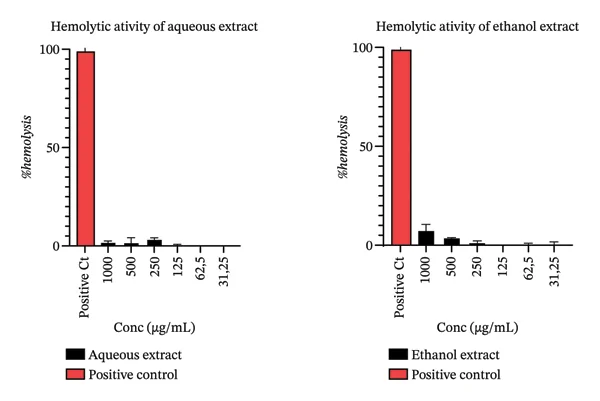
Hemolytic activity of aqueous and ethanol extracts of *T. superba* at different concentrations.

Figure 10 illustrates the cytotoxic effect of *T. superba* aqueous and ethanol extracts on human red blood cells (erythrocytes). Hemolysis at high concentrations of 500 μg/mL and 1000 μg/mL for the ethanolic and aqueous extracts was 3.6 ± 0.28%, 7.6 ± 2.96%, and 1.8 ± 2.36%, 2 ± 0.64%, respectively, proving that the aqueous extract was comparatively less cytotoxic compared to the ethanolic extract. The ethanolic extract showed higher hemolytic activity than the aqueous extract, but both extracts showed hemolysis less than 10% at concentrations studied, indicating relatively low cytotoxicity. The positive control was 1.0% Tween 20, which showed 99.15% hemolysis in both cases. There was a dose‐dependent increase in red blood cell lysis by the extracts. All treatments were done in triplicate.

### 3.8. Qualitative Phytochemical Content of *T. superba* Extracts

Preliminary phytochemical screening of the ethanolic and aqueous extracts of *T. superba* showed that both extracts contained flavonoids, phenols, and tannins. Notably, alkaloids were found only in the ethanolic extract, while quinones were present exclusively in the aqueous extract. Triterpenoids were not detected in either extract (Table 2).

**TABLE 2:** Some phytochemical constituents present in the bark extracts of *T. superba*.

Constituents	Aqueous extract	Ethanol extract
Alkaloids	−	+
Flavonoids	+	+
Phenols	+	+
Triterpenoids	−	−
Quinones	+	−
Tannins	+	+

*Note:* (+) present, (−) absent.

### 3.9. UHPLC‐MS/MS Analysis

**FIGURE 11:**
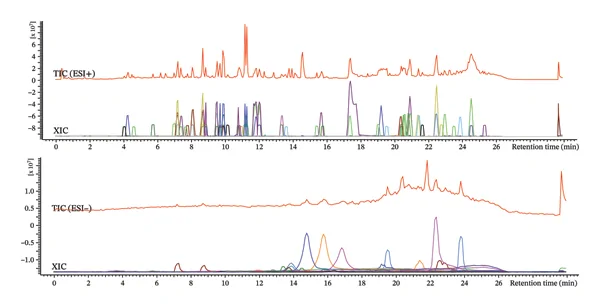
UHPLC‐DAD‐ESI chromatograms with TIC and XIC in positive and negative modes.

UHPLC‐MS/MS screening was performed to obtain the metabolite profiles of the bark extracts of *T. superba*, and qualitative analysis enabled the putative annotation of 82 major characteristic peaks (Figure 11). The complete chemical fingerprint of the extracts, corresponding to these major peaks, is presented in Table 3, which collectively demonstrates the abundant phytochemical composition of *T. superba*.

**TABLE 3:** Phytochemical constituents of *T. superba* bark extract as revealed by UHPLC‐MS/MS.

NO.	Comp.	tR (min)	Formula	Ion mode	Cal. m/z	Obs. m/z	Diff. (ppm)	Fragments	Area
1	Arjunone	1.36	C_19_H_20_O_6_	[M+H]^+^	345.1338	345.1329	−2.61	294.0824, 146.0559, 137.0595, 329.0229, 230.8873, 191.0678	0.02%
2	Isotretinoin	1.44	C_27_H_22_O_18_	[M+H]^+^	635.0884	635.0872	−1.89	345.0223, 581.0538, 616.6631, 465.0599, 301.0289	0.02%
3	Arjunin	1.48	C_41_H_26_O_26_	[M+H]^+^	935.0791	935.0769	−2.35	633.0721, 505.0361, 615.0556, 493.0505, 469.9863	0.05%
4	[(2*S*,3*S*,6*R*)‐2‐methoxy‐6‐methyl‐4‐oxooxan‐3‐yl] 2,4‐dihydroxy‐6‐methylbenzoate	1.70	C_15_H_18_O_7_	[M+H]^+^	311.1131	311.1119	−3.86	209.1177, 237.1120, 151.0406, 174.0661, 137.0589, 220.9905	2.78%
5	Punicalin	1.87	C_34_H_22_O_22_	[M+H]^+^	783.0681	783.0661	−2.55	766.0612, 621.0158, 120.0522, 724.9571, 577.0236, 749.0953	0.05%
6	Gemin D	2.95	C_27_H_22_O_18_	[M+H]^+^	635.0884	635.0874	−1.57	310.0978, 447.0522, 277.0733, 122.0703, 387.0297, 578.9844	0.03%
7	Luteolin	3.12	C_15_H_10_O_6_	[M+H]^+^	287.0556	287.0551	−1.74	115.0866, 157.0961, 175.1071	0.14%
8	Acutissimin A	3.51	C_56_H_38_O_31_	[M+H]^+^	1207.1475	1207.1420	−4.56	1055.1034, 753.0907, 163.0422, 1191.1339, 829.9766, 710.1137	0.02%
9	4‐Hydroxybenzoic acid	3.74	C_7_H_6_O_3_	[M+H]^+^	139.0395	139.0398	2.16	123.0182, 126.0298, 100.9909, 108.9644	0.05%
10	Homovanillic acid	3.74	C_9_H_10_O_4_	[M+H]^+^	183.0657	183.0648	−4.92	139.0829, 109.0253, 124.0446, 151.2403	0.01%
11	Hesperetin	3.74	C_16_H_14_O_6_	[M+H]^+^	303.0869	303.0859	−3.30	153.0531, 286.2175, 138.0291, 163.0372, 122.0335, 109.0618, 259.0592, 220.9155	0.18%
12	Methyl 3,4,5‐trihydroxy‐2‐ (6,7,13,14‐tetrahydroxy‐3,10‐dioxo‐2,9‐dioxatetracyclo [6.6.2.04,16.011,15]hexadeca‐1 (15),4 (16),5,7,11,13‐hexaen‐5‐yl)benzoate	3.85	C_22_H_12_O_13_	[M+H]^+^	485.0356	485.0333	−4.74	453.0076, 103.0358, 193.0448, 363.0194, 407.0035, 167.0333	2.56%
13	Brevifolin‐carboxylic acid	4.08	C_13_H_8_O_8_	[M+H]^+^	293.0297	293.0288	−3.07	219.0287, 247.0239, 233.0071, 191.0333	0.06%
14	Cerasidin	4.25	C_19_H_20_O_6_	[M+H]^+^	345.1338	345.1336	−0.58	137.0608, 171.0456, 151.0743, 283.0937, 328.1712	0.11%
15	Tellimoside	4.25	C_28_H_24_O_16_	[M+H]^+^	617.1143	617.1125	−2.92	163.0419, 303.0875, 153.0185, 439.0936, 345.0266, 245.0818, 205.0479	0.15%
16	Casuarictin	4.25	C_41_H_28_O_26_	[M+H]^+^	937.0947	937.0940	−0.75	345.0233, 411.0324, 153.0185, 277.0329, 617.0791, 255.0489	0.11%
17	Oleanic acid	4.31	C_30_H_48_O_3_	[M+H]^+^	457.3682	457.3672	−2.19	457.3670, 397.0097, 302.1085, 233.0097	0.01%
18	α‐Eleostearic acid	4.42	C_18_H_30_O_2_	[M+H]^+^	279.2324	279.2318	−2.15	109.1023, 137.1339, 121.1006, 209.1555, 173.1333, 100.0753	0.11%
19	Arjunic acid	4.48	C_30_H_48_O_5_	[M+H]^+^	489.3580	489.3578	−0.41	425.3414, 439.3204, 185.1318, 393.3124	5.19%
20	Chavibetol	4.76	C_10_H_12_O_2_	[M+H]^+^	165.0916	165.0912	−2.42	105.0695, 137.0598, 133.0636, 122.0348	0.04%
21	Flavogallonic acid	4.81	C_21_H_10_O_13_	[M+H]^+^	471.0200	471.0190	−2.12	453.0082, 166.9966, 287.0172, 302.0046	8.22%
22	Terminalin	5.32	C_28_H_10_O_16_	[M+H]^+^	603.0047	603.0033	−2.32	300.9968, 189.0195, 563.0480, 412.5339, 497.0033, 250.1171	0.19%
23	Potentillin	5.83	C_41_H_28_O_26_	[M+H]^+^	937.0947	937.0941	−0.64	255.0502, 411.0342, 617.0768, 919.0855, 345.0256, 277.0343	0.06%
24	Eschweilenol C	5.89	C_20_H_16_O_12_	[M+H]^+^	449.0720	449.0707	−2.89	303.0139, 129.0534, 153.0194	0.18%
25	Ellagic acid	6.00	C_14_H_6_O_8_	[M+H]^+^	303.0141	303.0137	−1.32	275.0174, 201.0148, 285.0019, 135.0812, 214.0245	0.18%
26	(1R,4aR,6aR,6aS,6bR,8aR,9S,10R,11R,12aS,14bS)‐1,10,11‐trihydroxy‐9‐(hydroxymethyl)‐2,2,6a,6b,9,12a‐hexamethyl‐13‐oxo‐3,4,5,6,6a,7,8,8a,10,11,12,14b‐dodecahydro‐1H‐picene‐4a‐carboxylic acid	6.74	C_30_H_46_O_7_	[M−H]^−^	517.3165	517.3159	−1.16	417.1740, 487.3213, 394.8680, 344.8664, 96.8590	0.01%
27	Combretastatin	6.97	C_18_H_22_O_6_	[M+H]^+^	335.1495	335.1484	−3.28	153.0544, 271.0960, 239.0706, 137.0604	5.71%
28	Butyl 2‐ethylhexyl phthalate	6.97	C_20_H_30_O_4_	[M+H]^+^	335.2222	335.2215	−2.09	189.1668, 105.0685, 145.0607, 229.0559	0.02%
29	3‐O‐Methylducheside A	7.19	C_21_H_18_O_12_	[M+H]^+^	463.0877	463.0858	−4.10	331.0453, 299.0190, 316.0213	5.31%
30	7‐H‐MDF	7.36	C_16_H_14_O_4_	[M+H]^+^	271.0970	271.0963	−2.58	151.0375, 107.0484, 214.2120, 117.0682	0.16%
31	Scutellarein	7.76	C_15_H_10_O_6_	[M+H]^+^	287.0556	287.0546	−3.48	255.0657, 271.0984, 227.0698, 161.0253	0.07%
32	Pyrogallol	8.32	C_6_H_6_O_3_	[M+H]^+^	127.0395	127.0393	−1.57	110.0581, 100.0760	0.08%
33	(4a*S*,6a*R*,6a*S*,6b*R*,8a*R*,9*R*,10*R*,11*R*,12a*S*)‐10,11‐dihydroxy‐9‐(hydroxymethyl)‐2,2,6a,6b,9,12a‐hexamethyl‐13‐oxo‐4,5,6,6a,7,8,8a,10,11,12‐decahydro‐3H‐picene‐4a‐carboxylic acid	8.32	C_30_H_44_O_6_	[M+H]^+^	501.3216	501.3207	−1.80	483.3100, 465.2991, 469.3321, 201.1629, 411.2904, 187.1472, 419.2927, 263.1636	0.28%
34	Arjunglucoside III	8.55	C_36_H_56_O_11_	[M+H]^+^	665.3901	665.3892	−1.35	503.3359, 316.0921, 332.0461, 153.0178, 439.3195	0.07%
35	3,3′‐Di‐O‐methylellagic acid	8.66	C_16_H_10_O_8_	[M+H]^+^	331.0454	331.0442	−3.62	299.0161, 316.0185, 169.0855	6.34%
36	2′,4′,5,7‐Tetramethoxy‐8‐methylflavanone	8.72	C_20_H_22_O_6_	[M+H]^+^	359.1495	359.1490	−1.39	327.1209, 253.0875, 281.1144, 313.1065, 149.0207, 189.0919	0.20%
37	(3*R*,4a*R*,6a*R*,6a*S*,6b*R*,8a*R*,12a*S*)‐3‐hydroxy‐2,2,6a,6b,9,9,12a‐heptamethyl‐10‐oxo‐3,4,5,6,6a,7,8,8a,11,12‐decahydro‐1H‐picene‐4a‐carboxylic acid	8.83	C_30_H_44_O_4_	[M+H]^+^	469.3318	469.3306	−2.56	205.1572, 201.1624, 451.3185, 423.3244, 247.1675, 405.3143, 147.1164	11.21%
38	2*α*,3*α*,24‐Trihydroxyolean‐11,13 (18)‐dien‐28‐oic acid	8.83	C_30_H_46_O_5_	[M+H]^+^	487.3424	487.3412	−2.46	187.1472, 423.3264, 469.3318, 451.3211, 201.1648, 405.3158	2.62%

39	Maslinic lactone	8.89	C_30_H_48_O_6_	[M+H]^+^	505.3529	505.3525	−0.79	469.3288, 341.1379, 205.1596, 424.3278, 452.3216, 411.2899, 199.1474, 187.1473	0.28%
				[M−H]^−^	503.3373	503.3372	−0.20	441.3588, 338.9288, 277.9363, 250.6937, 222.9758, 62.0376	
40	Combretastatin A‐1	9.12	C_18_H_20_O_6_	[M+H]^+^	333.1338	333.1330	−2.40	273.0761, 131.0488, 301.1054, 269.0787, 161.0598, 167.0691, 153.0536	0.13%
41	Olean‐12‐en‐28‐oic acid, 2,3,19‐trihydroxy‐23‐[(3,4,5‐trihydroxybenzoyl)oxy]‐, *β*‐D‐glucopyranosyl ester, (2*α*,3*β*,4*α*,19*α*)‐ (ACI)	9.34	C_43_H_62_O_15_	[M+H]^+^	819.4167	819.4166	−0.12	783.3919, 801.4052, 469.3262, 315.0736, 163.1490, 439.2105, 621.3438	0.03%
42	2,2‐Dimethyl‐2H‐chromene‐6‐carboxylic acid	9.46	C_12_H_12_O_3_	[M+H]^+^	205.0865	205.0859	−2.93	175.0325, 143.0496, 121.0650, 115.0528, 190.0608	0.21%
43	Phomochromone B	9.46	C_12_H_14_O_4_	[M+H]^+^	223.0970	223.0962	−3.59	205.0847, 177.0895, 146.0717, 161.0586, 190.0621, 115.0536	0.08%
44	Carda‐16,20 (22)‐dienolide, 3‐[(6‐O‐*β*‐D‐glucopyranosyl‐*β*‐D‐galactopyranosyl)oxy]‐14‐hydroxy‐, (3*β*,5*β*)‐ (9CI)	9.77	C_35_H_52_O_14_	[M−H]^−^	695.3279	695.3287	1.15	423.8628, 383.8060, 487.3450, 596.7895, 159.0453, 147.0193	0.09%
45	Arjunolic acid	9.91	C_30_H_48_O_5_	[M+H]^+^	489.3580	489.3568	−2.45	205.1588, 407.3321, 247.1699, 163.1469	5.20%

46	Arjungenin	10.08	C_30_H_48_O_6_	[M+H]^+^	505.3529	505.3521	−1.58	451.3252, 405.3152, 469.3006, 424.3287, 205.1581, 199.1472, 151.0739, 133.1024	0.09%
				[M−H]^−^	503.3373	503.3372	−0.20	167.0524, 334.8308, 355.9561, 456.3330486.3052, 147.0195	
47	Olean‐12‐en‐28‐oic acid, 3‐[(3‐O‐*β*‐D‐galactopyranosyl‐*β*‐D‐glucopyranosyl)oxy]‐2,19‐dihydroxy‐, (2*α*,3*β*,19*α*)‐ (9CI)	10.19	C_42_H_68_O_15_	[M+H]^+^	813.4637	813.4642	0.61	479.1706, 331.1517, 440.3548, 661.2674, 453.3372, 313.1435	0.03%
48	Kaempferol	10.93	C_15_H_10_O_6_	[M+H]^+^	287.0556	287.0543	−4.53	287.0532, 255.1000, 240.0751, 337.0687	2.54%
49	Olean‐12‐en‐28‐oic acid, 2,3,6‐trihydroxy‐23‐[(3,4,5‐trihydroxybenzoyl)oxy]‐, *β*‐D‐glucopyranosyl ester, (2*α*,3*β*,4*α*,6*β*)‐ (ACI)	11.04	C_43_H_62_O_15_	[M+H]^+^	819.4167	819.4159	−0.98	657.3632, 783.3885, 631.3829, 247.1682, 204.1903	0.03%
50	Lucyoside B	11.15	C_42_H_68_O_15_	[M+H]^+^	813.4637	813.4658	2.58	453.3366, 471.3450, 489.3557	2.61%
51	Hyptatic acid A	11.21	C_30_H_48_O_5_	[M+H]^+^	489.3580	489.3588	1.63	489.3533, 425.3338, 107.0466, 203.1754	0.35%
52	*β*‐D‐Glucopyranosyl (2*α*,3*β*)‐2,3‐dihydroxyursa‐12,18‐dien‐28‐oate	11.29	C_36_H_56_O_9_	[M+H]^+^	633.4003	633.4021	2.84	355.1476, 271.2451, 284.9838	0.12%
53	Arjunetin	11.33	C_36_H_58_O_10_	[M+H]^+^	651.4108	651.4098	−1.54	453.3360, 435.3254, 407.3304, 471.3460, 125.0949, 247.1686, 187.1468, 201.1621	4.96%
54	(4a*S*,6a*S*,6b*R*,9*R*,10*R*,11*R*,12a*R*)‐10,11‐dihydroxy‐2,2,6a,6b,9,12a‐hexamethyl‐9‐[(3,4,5‐trihydroxybenzoyl)oxymethyl]‐1,3,4,5,6,6a,7,8,8a,10,11,12,13,14b‐tetradecahydropicene‐4a‐carboxylic acid	11.49	C_37_H_52_O_9_	[M+H]^+^	641.3690	641.3686	−0.62	453.3356, 407.3308, 435.3259, 187.1478, 177.1638, 261.1841	0.25%
55	Olean‐12‐en‐28‐oic acid, 2,3,19‐trihydroxy‐23‐[(3,4,5‐trihydroxybenzoyl)oxy]‐, (2*α*,3*β*,4*α*,19*α*)‐ (ACI)	11.55	C_37_H_52_O_10_	[M+H]^+^	657.3639	657.3633	−0.91	469.3289, 621.3439, 451.3199, 640.3575, 177.1638, 405.3114	0.05%
56	Olean‐12‐en‐28‐oic acid, 2,3‐dihydroxy‐23‐[(3,4,5‐trihydroxybenzoyl)oxy]‐, *β*‐D‐glucopyranosyl ester, (2*α*,3*β*,4*α*)‐ (ACI)	11.59	C_43_H_62_O_14_	[M+H]^+^	803.4218	803.4223	0.62	315.0711, 453.3372, 153.0189, 279.0499, 297.0571	0.17%
57	Anolignan B	11.61	C_18_H_18_O_2_	[M+H]^+^	267.1385	267.1380	−1.87	121.0648, 107.0495, 135.0798, 173.0964, 207.0986	0.05%
58	(−)‐Epicatechin	11.66	C_15_H_14_O_6_	[M+H]^+^	291.0869	291.0856	−4.47	139.0387, 123.0435, 207.0652, 237.1127, 147.0442161.0606	5.19%
59	Tetradecylamine	12.23	C_14_ H_31_N	[M+H]^+^	214.2535	214.2528	−3.27	198.0504, 155.0001, 129.9720, 138.9936, 145.0707	0.05%
60	6,7,13,14‐tetramethoxy‐2,9‐dioxatetracyclo[6.6.2.04,16.011,15]hexadeca‐1 (15),4,6,8 (16),11,13‐hexaene‐3,10‐dione	12.34	C_18_H_14_O_8_	[M+H]^+^	359.0767	359.0758	−2.51	286.0101, 270.0146, 329.0285, 300.0235, 115.0540, 217.0474, 155.0486	0.20%
61	Termilignan	12.46	C_19_H_20_O_3_	[M+H]^+^	297.1491	297.1479	−4.04	135.0439, 121.0271, 171.0793	6.72%
62	Termilignan B	13.30	C_19_H_18_O_3_	[M+H]^+^	295.1334	295.1318	−5.42	280.2639, 187.0762, 249.1268, 121.0639	2.93%
63	2‐Aminooctadecane‐1,3,4‐triol	13.36	C_18_H_39_NO_3_	[M+H]^+^	318.3008	318.2998	−3.14	300.2899, 282.2790, 270.2786, 264.2690, 252.2682	5.23%

64	Bayogenin	13.47	C_30_H_48_O_5_	[M+H]^+^	489.3580	489.3577	−0.61	201.1643, 453.3351, 407.3293, 425.3406, 189.1641, 471.3502	0.16%
				[M−H]^−^	487.3424	487.3439	3.08	469.3441, 425.3591	
65	Benzoic acid, 3‐methoxy‐4‐[[6‐O‐(3,4,5‐trihydroxybenzoyl)‐*β*‐D‐glucopyranosyl]oxy]‐ (9CI, ACI)	13.70	C_21_H_22_O_13_	[M−H]^−^	481.0982	481.0971	−2.29	147.0193, 396.1732, 311.2211, 62.0395	0.20%
66	Geranyl tiglate	14.32	C_15_H_24_O_2_	[M+H]^+^	237.1855	237.1848	−2.95	124.0521, 191.1064, 111.0795, 157.8950, 181.0507	0.04%
67	CHEMBL3590461	14.38	C_21_ H_37_N	[M+H]^+^	304.3004	304.2997	−2.30	212.2364, 154.2632, 180.8658, 194.1185, 189.1259	0.17%
68	(2*α*,3*β*)‐2,3‐Dihydroxyursa‐12,20 (30)‐dien‐28‐oic acid	14.55	C_30_H_46_O_4_	[M+H]^+^	471.3474	471.3458	−3.39	407.3322, 205.1590, 201.1628, 147.1162, 425.3419, 187.1485	5.48%
69	4‐[(2*R*,3*S*,4*R*,5*S*)‐5‐(4‐methoxyphenyl)‐3,4‐dimethyloxolan‐2‐yl]phenol	14.83	C_19_H_22_O_3_	[M+H]^+^	299.1647	299.1643	−1.34	121.0759, 107.0464, 133.1022, 284.2926, 281.1572, 224.1491, 211.1488, 165.0918	0.07%
70	Cyanidin	17.15	C_15_H_11_O_6_	[M]^+^	287.0556	287.0548	−2.79	243.0292, 121.0279, 233.0395, 213.0514	0.06%
71	Methyl gallate	17.49	C_8_H_8_O_5_	[M+H]^+^	185.0450	185.0443	−3.78	153.0181, 125.0227, 166.9433, 141.9206, 102.9468	0.02%
72	*α*‐Caryophyllene alcohol	18.40	C_15_ H_26_O	[M+H]^+^	223.2062	223.2055	−3.14	125.0722, 150.1055, 107.0860, 165.8385, 118.1128, 131.0808	0.08%
73	(Z)‐2‐((Z)‐hexadec‐7‐enyl)‐3‐methylbut‐2‐enedioic acid	18.91	C_21_H_36_O_4_	[M+H]^+^	353.2692	353.2686	−1.70	290.1483, 107.0859, 336.2874, 309.2431, 266.1585	0.08%
74	Olean‐12‐en‐28‐oic acid, 2,3,6,23‐tetrahydroxy‐, *β*‐D‐glucopyranosyl ester, (2*α*,3*β*,4*α*,6*β*)‐ (9CI, ACI)	18.96	C_36_H_58_O_11_	[M−H]^−^	665.3901	665.3915	2.10	116.9815, 147.0198, 551.8817, 585.4008, 333.2151	0.16%
75	Ursolic acid	19.42	C_30_H_48_O_3_	[M+H]^+^	457.3682	457.3672	−2.19	411.3617, 191.1788, 439.3553, 203.1783, 249.1812	0.11%
76	(2*R*,3*S*,4*R*,5*S*)‐2,5‐bis(4‐methoxyphenyl)‐3,4‐dimethyloxolane	20.38	C_20_H_24_O_3_	[M+H]^+^	313.1804	313.1807	0.96	295.1781, 129.0689, 153.0190, 137.0599	2.81%
77	Flavan	20.83	C_15_ H_14_O	[M+H]^+^	211.1123	211.1119	−1.89	178.0765, 151.0529, 191.0860, 195.0820, 115.0527, 127.0553	0.11%
78	Coumarin	27.05	C_9_H_6_O_2_	[M+H]^+^	147.0446	147.0441	−3.40	121.0638, 119.0494, 117.9917, 103.0349, 111.9607, 130.9779	0.04%
79	4‐Deoxythreonic acid	27.33	C_4_H_8_O_4_	[M+H]^+^	121.0501	121.0503	1.65	121.0509, 103.0544	0.31%
80	Vanillic acid	27.42	C_8_H_8_O_4_	[M+H]^+^	169.0501	169.0499	−1.18	123.0437, 150.9470, 111.0432, 134.0448, 139.0407	0.02%
81	Stigmasterol	28.50	C_29_ H_48_O	[M+H]^+^	413.3783	413.3775	−1.94	371.2588, 294.1827, 241.0327, 255.2145, 149.1313	0.02%
82	Kynurenic acid	28.87	C_10_H_7_NO_3_	[M+H]^+^	190.0504	190.0498	−3.16	116.0494, 144.0411	0.04%

### 3.10. Quantitative Phytochemical Content of *T. superba* Extracts

**FIGURE 12:**
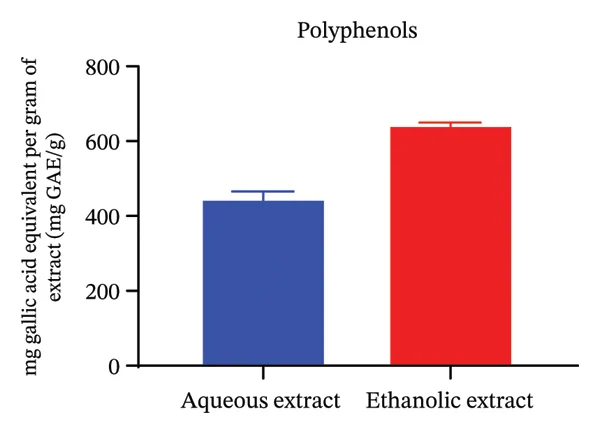
Total phenolic contents of the bark extracts of *T*. *superba*. Values are expressed as mean ± SD (*n* = 8). Differences between extracts were statistically significant (*p* < 0.001).

**FIGURE 13:**
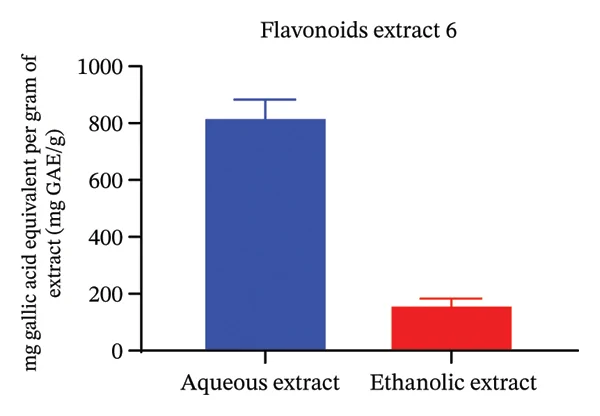
Total flavonoid contents of the bark extracts of *T*. *superba*. Values are expressed as mean ± SD (*n* = 8). Differences between extracts were statistically significant (*p* < 0.001).

Figures 12 and 13 show the total phenol and flavonoid contents of the aqueous and ethanolic extracts. The ethanolic extract exhibited a higher total phenolic content (641.08 ± 8.90 mg GAE/g) than the aqueous extract (443.96 ± 21.57 mg GAE/g). The ethanolic extract exhibited a significantly higher total phenolic content. The ethanolic extract exhibited a significantly higher total phenolic content compared to the aqueous extract (*p* < 0.001). Total flavonoid content was significantly higher in the aqueous extract (817.50 ± 65.22 mg GAE/g) than in the ethanol extract (159.17 ± 24.02 mg GAE/g), *p* < 0.001. Error bars show the standard deviation of eight replicate measurements (*n* = 8).

### 3.11. Anthelminthic Molecular Docking Analysis: An In Silico Approach

**FIGURE 14:**
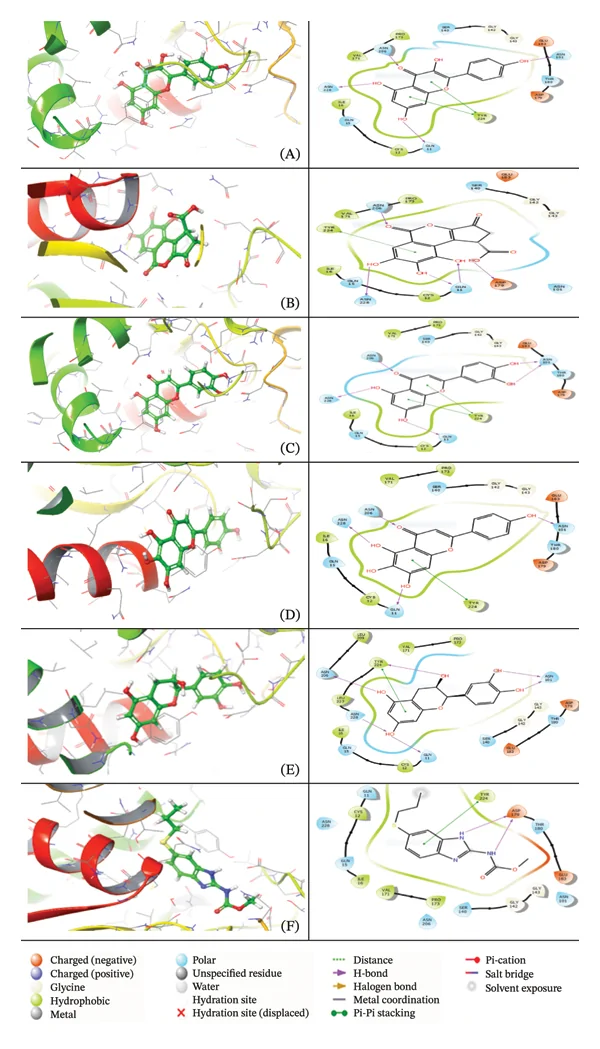
3D and 2D interactions of ligands of *T. superba* and the *β*‐tubulin protein. (A) Kaempferol, (B) brevifolin‐carboxylic acid, (C) luteolin, (D) scutellarein, (E) (−)‐epicatechin, (F) albendazole.

**FIGURE 15:**
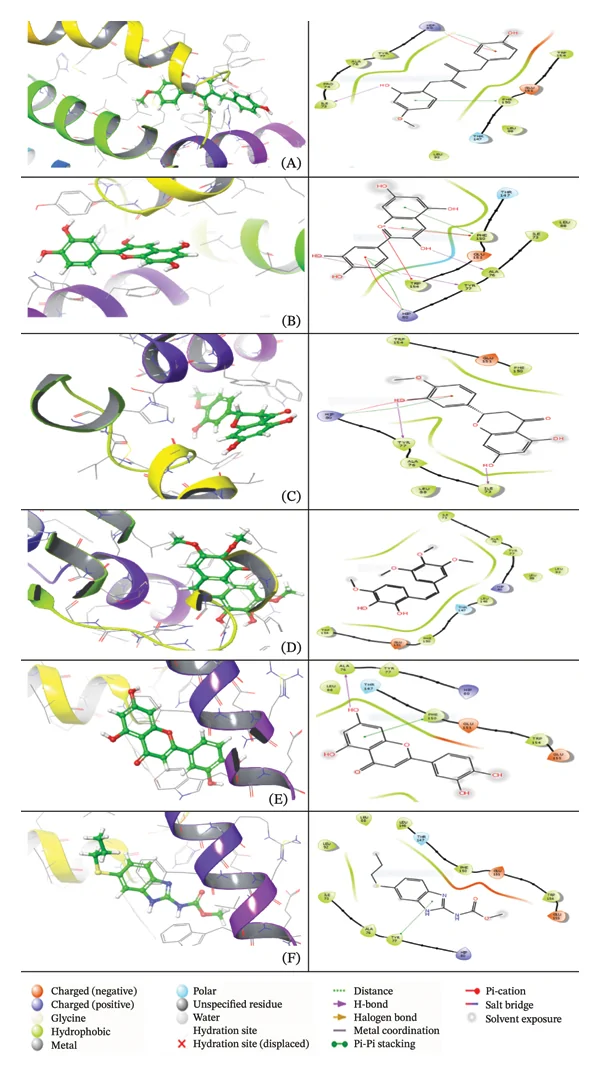
3D and 2D interactions of ligands of *T. superba* and SDH protein. (A) Termilignan, (B) cyanidin, (C) hesperidin, (D) combretastatin A‐1, (E) luteolin, (F) albendazole.

Molecular docking of the 82 identified compounds against *β*‐tubulin and SDH revealed varying binding affinities as reflected by their docking scores, with albendazole serving as a positive control. Table 4 displays the docking scores of five top ligands for both helminth protein targets. Kaempferol showed the strongest binding affinity toward *β*‐tubulin with the lowest docking score (−8.033 kcal/mol), followed by brevifolin‐carboxylic acid (−7.985 kcal/mol), luteolin (−7.954 kcal/mol), scutellarein (−7.373 kcal/mol), and (−)‐epicatechin (−6.980 kcal/mol). These tested phytochemicals exhibited markedly better binding affinities than the reference drug albendazole (−3.728 kcal/mol), indicating potentially stronger theoretical interactions with the target protein. Meanwhile, termilignan showed the strongest binding affinity toward SDH with the lowest docking score (−6.767 kcal/mol), followed by cyanidin (−6.659 kcal/mol), hesperidin (−6.314 kcal/mol), combretastatin A‐1 (−6.274 kcal/mol), and luteolin (−6.166 kcal/mol). These tested phytochemicals exhibited higher binding affinities than the reference drug albendazole (−5.541 kcal/mol), suggesting potentially stronger theoretical interactions with the target enzyme. Figures 14 and 15 shows the interactions between ligands and the receptor proteins of *β*‐tubulin and SDH.

**TABLE 4:** Docking scores of ligands of *T. superba* with *β*‐tubulin and SDH.

**No**	**Compound**	**Docking score with *β*-tubulin**	**Glide emodel**

1	**Kaempferol**	−8.033	−66.384
2	**Brevifolin-carboxylic acid**	−7.985	−63.983
3	**Luteolin**	−7.954	−70.553
4	**Scutellarein**	−7.373	−62.094
5	**(−)-Epicatechin**	−6.980	−64.153
Positive control	**Albendazole**	−3.728	−42.294

**No**	**Compound**	**Docking score with SDH**	**Glide emodel**

1	**Termilignan**	−6.767	−44.722
2	**Cyanidin**	−6.659	−46.860
3	**Hesperidin**	−6.314	−46.048
4	**Combretastatin A-1**	−6.274	−41.861
5	**Luteolin**	−6.166	−42.848
Positive control	**Albendazole**	−5.541	−44.858

For the *β*‐tubulin protein, the target proteins and ligand interactions are shown in Figure 14. Kaempferol interacts through H‐bonds with amino acids such as Asparagine101, Asparagine228, Asparagine206, and Glutamine11. Also, it interacts through pi–pi stacking with Tyrosine224. Its activity could be due to its abundant OH‐groups and 3 benzene rings. Brevifolin‐carboxylic acid interacts via H‐bonds with amino acids such as Asparagine206, Asparagine228, Glutamine11, and Aspartic acid179. Pi–pi stacking occurs with tyrosine (224). Its inhibitory activity could be due to the presence of its abundant carbonyl groups. Luteolin interacts through H‐bonds with Asparagine228, Asparagine206, Asparagine101, and Glutamine11. Scutellarein inhibits the protein by interacting with Asparagine228, Asparagine206, Asparagine101, and Glutamine11. (−)‐Epicatechin interacts with proteins such Asparagine206, Tyrosine224, Asparagine228, Glutamine11, and Asparagine101 through H‐bonds. Albendazole (positive control) engages with the target protein through a single H‐bond with the amino acid being Aspartic acid179. The inhibitory ability of albendazole could be due to the presence of its many amino groups.

For the SDH protein, interactions between the ligands and target proteins present termilignan as the most reactive ligand, interacting through H‐bonds with the amino acid Isoleucine73. Pi–pi stacking occurs with Phenylalanine150 and HIP80. Cyanidin engages through H‐bonds with Tyrosine77 and Alanine76. Pi–cation interaction occurs with Phenylalanine150, Tryptophan154, and HIP(80). While pi–pi stacking is with Phenylalanine150, Tyrosine154, and HIP80. Hesperidin interacts via H‐bonds with Tyrosine77 and Isoleucine73. Combretastatin A‐1, the fourth most reactive compound, shows no visible interactions with amino acids. Luteolin interacts via H‐bond with Alanine76, and pi–pi stacking is with Phenylalanine 150. Albendazole acting as a positive control ligand interacts through pi–pi stacking interaction only with Tyrosine77.

### 3.12. *In Silico* ADME and Toxicity Predictions

The drug‐like properties of the tested ligands active against *β*‐tubulin and SDH proteins were highly favorable (Tables 5 and 6). The compounds generally showed very good pharmacokinetic and bioavailability properties compared to albendazole. There was a zero violation of the Lipinski’s rule of five for all tested compounds, and these compounds generally showed good intestinal and oral absorption properties. All the tested compounds generally showed minimal toxicity for the tested target organs (Tables 7 and 8). All compounds were inactive for cytotoxicity and for hepatotoxicity except albendazole, which presented a 0.78 probability of hepatotoxicity. A majority of the compounds were nonmutagenic. (−)‐Epicatechin showed the highest predicted lethal dose 50 (LD_50_ = 10,000 mg/kg), the only compound in the predicted toxicity class 6 (PTC = 6), followed by cyanidin with LD_50_ = 5000 mg/kg and PTC = 5.

**TABLE 5:** ADME predictions for compounds active against *β*‐tubulin protein.

No	Compound	% human oral absorption	QPlogKhsa	QplogHerg	QPPCaco	QPlogBB	Rule of five	Rule of three	QPlogPo/w	PSA	QPlogS
1	**Kaempferol**	63.637	−0.191	−5.201	51.240	−1.893	0	0	1.041	121.6	−3.157
2	**Brevifolin-carboxylic acid**	24.522	−0.879	−1.789	1.698	−2.476	0	1	−1.116	177.9	−1.880
3	**Luteolin**	61.516	−0.204	−5.011	42.593	−1.933	0	0	0.924	120.5	−3.041
4	**Scutellarein**	63.231	−0.201	−5.042	50.401	−1.861	0	0	0.993	119.6	−3.044
5	**(−)-Epicatechin**	60.584	−0.412	−4.732	53.201	−1.882	0	1	0.469	115.9	−2.616
6	**Albendazole**	93.271	0.230	−5.195	601.623	−0.886	0	0	2.819	76.395	−4.570

**TABLE 6:** ADME predictions for compounds active against SDH protein.

N^0^	Compound	% Human oral absorption	QPlogKhsa	QplogHerg	QPPCaco	QPlogBB	Rule of five	Rule of three	QPlogPo/w	PSA	QPlogS
1	**Termilignan**	100.000	0.473	−5.494	1276.809	−0.806	0	0	4.293	51.186	−4.607
2	**Cyanidin**	—	—	—	—	—	—	—	—	—	—
3	**Hesperidin**	75.239	0.009	−5.017	130.638	−1.533	0	0	1.780	108.4	−4.252
4	**Combretastatin A-1**	100.000	0.171	−4.527	1339.870	−0.829	0	0	3.311	73.002	−4.821
5	**Luteolin**	61.516	−0.204	−5.011	42.593	−1.933	0	0	0.924	120.5	−4.073
6	**Albendazole**	100.000	0.638	−5.184	574.140	−0.971	0	0	3.862	76.252	−5.328

**TABLE 7:** Toxicity predictions for compounds active against *β*‐tubulin protein.

N^0^	Chemical compound	Predicted LD50 (mg/Kg)	PTC/6	Hepato./Proba.	Cyto./Proba.	Neuro./Proba.	Nephro/Poba.	BBB/Proba.	Immuno/Proba	Respi/Proba	Cardio/Proba	Mutagen/Proba
1	**Kaempferol**	3919	5	IA (0.68)	IA (0.98)	IA (0.89)	A (0.62)	A (0.57)	IA (0.96)	A (0.83)	IA (0.91)	IA (0.52)
2	**Brevifolin-carboxylic acid**	1000	4	IA (0.76)	IA (0.72)	IA (0.91)	A (0.63)	A (0.64)	IA (0.98)	A (0.81)	IA (0.89)	IA (0.50)
3	**Luteolin**	3919	5	IA (0.69)	IA (0.99)	IA (0.89)	A (0.62)	A (0.53)	IA (0.97)	A (0.83)	IA (0.99)	A (0.51)
4	**Scutellarein**	3919	5	IA (0.69)	IA (0.99)	IA (0.89)	A (0.62)	A (0.53)	IA (0.99)	A (0.83)	IA (0.99)	A (0.51)
5	**(−)-Epicatechin**	10,000	6	IA (0.72)	IA (0.84)	IA (0.90)	A (0.62)	A (0.55)	IA (0.96)	A (0.79)	IA (0.99)	IA (0.55)
6	**Albendazole**	970	4	A (0.78)	IA (0.75)	A (0.59)	A (0.54)	A (0.89)	IA (0.84)	A (0.52)	IA (0.80)	IA (0.71)

*Note:* A = active, IA = inactive, Proba = probability, Hepato = hepatotoxicity, Cyto = cytotoxicity, Neuro = neurotoxicity, Nephro = nephrotoxicity, Respi = respiratory toxicity, Immuno = immunotoxicity, Cardio. = cardiotoxicity, mutagen = mutagenicity.

Abbreviations: BBB = blood–brain barrier, PTC = predicted toxicity class.

**TABLE 8:** Toxicity predictions for compounds active against SDH protein.

N^0^	Chemical compound	Predicted LD50 (mg/kg)	PTC/6	Hepato./proba.	Cyto./proba.	Neuro./proba.	Nephro/poba.	BBB/proba.	Immuno/proba	Respi/proba	Cardio/proba	Mutagen/proba
1	**Termilignan**	913	4	IA (0.68)	IA (0.85)	IA (0.59)	A (0.52)	A (0.52)	A (0.66)	IA (0.56)	IA (0.78)	IA (0.76)
2	**Cyanidin**	5000	5	IA (0.72)	IA (0.94)	IA (0.90)	A (0.58)	A (0.56)	IA (0.93)	A (0.81)	IA (0.95)	IA (0.58)
3	**Hesperidin**	2000	4	IA (0.70)	IA (0.86)	IA (0.87)	A (0.67)	IA (0.50)	A (0.90)	A (0.86)	A (0.99)	IA (0.87)
4	**Combretastatin A-1**	1560	4	IA (0.69)	IA (0.97)	IA (0.76)	A (0.54)	A (0.53)	A (0.98)	IA (0.67)	IA (0.69)	IA (0.69)
5	**Luteolin**	3919	5	IA (0.69)	IA (0.99)	IA (0.89)	A (0.62)	A (0.53)	IA (0.97)	A (0.83)	IA (0.99)	A (0.51)
6	**Albendazole**	970	4	A (0.78)	IA (0.75)	A (0.59)	A (0.54)	A (0.89)	IA (0.84)	A (0.52)	IA (0.80)	IA (0.71)

*Note:* A = active, IA = inactive, Proba = probability, Hepato = hepatotoxicity, Cyto = cytotoxicity, Neuro = neurotoxicity, Nephro = nephrotoxicity, Respi = respiratory toxicity, Immuno = immunotoxicity, Cardio. = cardiotoxicity, mutagen = mutagenicity.

Abbreviations: BBB = blood–brain barrier, PTC = predicted toxicity class.

## 4. Discussion

There has been a growing interest and attention given to the domain of efficacy and safety of plant‐based treatment intervention alternatives by researchers and pharmaceutical companies. These growing studies link the efficacy of the plant extracts to their secondary metabolites [60, 61]. *T. superba* extracts have commonly been used by the local populations and traditional healers for the treatment of intestinal helminths and many other infections [27].

Both aqueous and ethanolic extracts were found to contain flavonoids, phenols, and tannins, while alkaloids were exclusive to the ethanolic extract and quinones to the aqueous extract. These compounds are well recognized for their antioxidant, anthelmintic, and cytotoxic properties, and their differential distribution between the two extracts provides a rational phytochemical basis for the distinct biological activity profiles observed across all assays [62–64]. The absence of alkaloids in the aqueous extract is in agreement with their higher solubility in organic solvents of intermediate polarity than in purely aqueous systems [65, 66]. Quinones, found exclusively in the aqueous extract, may contribute to antioxidant and anthelmintic potential through redox‐active electron transfer, enzyme inactivation, and disruption of parasite energy metabolism [66, 67]. The rich flavonoid and tannin content may also contribute to the traditional use of *T. superba* as an anthelmintic as these compounds interfere with parasite neuromuscular function and disrupt cuticle integrity, respectively [63, 68].

### 4.1. Anthelmintic Activity

To provide experimental validation of the anthelmintic potential of *T. superba* extracts, their in vitro activity against *H. polygyrus* egg hatching and larval development was evaluated at various concentrations, with results compared to the reference anthelmintic albendazole.

Egg hatching was not greatly suppressed by the extracts compared to the standard anthelmintic drug (albendazole), with the aqueous extract being comparatively more active (IC_50_: 1.2 mg/mL) than the ethanolic extract (IC_50_: 2.1 mg/mL). Similar results were obtained by Cedric et al. [36] with extracts of *Persea americana* seeds, who also had the aqueous extract being more active against hatching of the embryonated eggs of *H*. *polygyrus* than the ethanolic extract. Meanwhile, Noumedem et al. (2024) and Cedric et al. [30] had contrary results with the ethanolic extract being more active than the aqueous extract against the egg hatching of *H*. *polygyrus* eggs. The egg hatching inhibition was concentration dependent, with an almost 100% inhibition seen in the wells with the highest concentration. Similar results were observed by other authors [30, 36, 69–73]. These findings run counter to those of Carvalho et al. [74] who used oil derived from *Carapa guianensis* seeds to treat *Haemonchus contortus* and found no ovicidal or larvicidal activity at any tested concentrations.

The comparatively higher ovicidal activity of the aqueous extract can be linked to the presence of its secondary metabolites. This could be due to total flavonoid content, which was higher in the aqueous extract than the ethanolic extract, and the quinones, which were present in the aqueous extract but lacking in the ethanolic extract. The aqueous extract might have entered the eggshell more effectively than the ethanolic extract because the eggshell layers are hydrophilic. These extracts may have an ovicidal impact because the active phytochemicals enter the helminth’s egg shell and prevent the blastomeres from undergoing segmentation [75] and paralyze the embryo inside the egg, thus inhibiting egg hatching. Furthermore, the extracts might have prevented the formation of the lipid barrier, which provides the embryo its impermeability and shields it from various harmful impacts of the environment [30]. The overall low egg hatch inhibition of the extracts compared to albendazole could be attributed to the protective ability of the nematode egg shell to withstand adverse conditions. Nematode egg shells typically consist of three to five protective layers adapted to enhance embryo survival, comprising an inner lipid layer, a middle chitinous protein layer, and an outer lipoprotein layer, which collectively confer resistance to oxidants, reducing agents, detergents, desiccation, acids, bases, and proteolytic substances [76, 77]. This inherent chemical resistance of the egg shell likely posed a barrier to the penetration of the phytochemical compounds into the egg. Reduced compound penetration may therefore have limited their interaction with internal egg structures and metabolic targets. This structural barrier consequently accounts for the comparatively lower anthelmintic efficacy of both extracts against eggs relative to albendazole, which as a synthetic compound may possess superior membrane penetration properties.

The larvicidal activity of both extracts varied across developmental stages as reflected by their IC_50_ values. Against L1 larvae, the aqueous extract demonstrated superior activity (IC_50_: 0.3 mg/mL) compared to the ethanolic extract (IC_50_:1.7 mg/mL), representing a greater potency. In contrast, the ethanolic extract exhibited markedly stronger activity against L2 larvae (IC_50_: 0.09 mg/mL) compared to the aqueous extract (IC_50_: 1.3 mg/mL), showing a greater potency. Similarly, the ethanolic extract demonstrated greater larvicidal activity against L3 larvae (IC_50_: 0.8 mg/mL) compared to the aqueous extract (IC_50_: 2.6 mg/mL), indicating greater efficacy. These findings collectively indicate that while the aqueous extract was more potent against the early stage L1 larvae, the ethanolic extract demonstrated consistently superior activity against the more developed L2 and L3 larval stages.

The different activity of the two extracts on the different stages is consistent with their phytochemical composition, since the flavonoid‐rich aqueous extract might preferentially target early larval stages such as L1, while the broader phenolic and alkaloid content of the ethanolic extract confers greater penetration capacity against the thicker cuticles of more developed L2 and L3 larvae [63, 78]. These observations are different from those of Wabo et al. [79] who had an overall weak activity of the aqueous extract of *Dichrocephala integrifolia* against all the developmental stages of *H*. *polygyrus*; meanwhile, the ethanolic extract was highly efficient. The results are also incongruent with those of other authors [30, 72, 73, 80], who had higher larvicidal activities with the ethanolic extract of different plants for both L1 and L2 stages of *H*. *polygyrus*. However, the results are similar to those obtained by Cedric et al. [36] who had a higher L1 and L2 larvicidal activity for the aqueous extract. The results also show that the L2 larvae were more susceptible to the extracts than the L1 larvae, with the ethanolic extract (IC_50_: 0.09 mg/mL) being more active against the L2 stage with larvicidal activity not very different from that of the standard drug albendazole (IC_50_: 0.03). This observation is in agreement with that of other authors [36, 71, 72, 81] who equally had L2 larvae to be more sensitive than the L1 larval stage. According to these authors, the L1 stage just resulting from hatching still possesses energy reserves and food, which are exhausted with time as they develop and molt, making them more sensitive at L2. By the L2 stage, larvae have depleted their energy reserves and must actively feed, increasing their uptake of anthelmintic compounds. Higher metabolic activity at this stage may make them more susceptible to substances that disrupt key metabolic pathways, eventually causing paralysis or death.

Although the ethanolic extract showed greater potency against L3 larvae than the aqueous extract, its activity at this stage was markedly lower than that recorded against L2 larvae, suggesting comparatively greater L3 resistance. The progressive decline in larvicidal potency of the aqueous extract across all three stages further corroborates the increasing resistance of larvae to phytochemical compounds as development advances, with L3 larvae proving the most resistant stage overall. This activity was concentration dependent, suggesting that higher concentrations may introduce additional active larvicidal compounds capable of overcoming the inherent resistance of this larval stage. A similar observation was reported by Komtangi et al. [77], who recorded only 38% larvicidal activity against L3 larvae of *H. polygyrus* using an ethanolic extract, corroborating the comparatively low susceptibility of this developmental stage to plant‐derived anthelmintic compounds.

The low susceptibility of L3 larvae may be explained by two key biological characteristics of this developmental stage. First, L3 larvae are enclosed within a double cuticle sheath that provides a robust physical barrier, significantly reducing the ability of bioactive compounds to penetrate and reach internal targets [77]. Second, L3 larvae are nonfeeding and depend solely on water stored within their cuticle, resulting in very low metabolic demands and consequently fewer accessible biochemical targets for anthelmintic compounds to disrupt [82]. Together, these structural and physiological characteristics collectively account for the reduced efficacy of both extracts against this larval stage compared to earlier developmental stages.

The stronger ovicidal and L1 larvicidal activity of the aqueous extract may reflect its elevated flavonoid content, which is known to disrupt parasite membranes and impair early developmental stages of nematodes [83, 84]. In contrast, the phenolic‐rich ethanolic extract demonstrated greater efficacy against the more developed L2 and L3 larvae by impairing larval development through protein binding, membrane destabilization, and energy metabolism interference [15, 67, 85]. Collectively, these findings suggest that differences in phytochemical composition influence anthelmintic efficacy across different parasite developmental stages. Beyond their direct anthelmintic effects, the flavonoids identified in both extracts may additionally confer cytoprotective benefits to the host by stabilizing cellular membranes and minimizing oxidative damage to host tissues [86].

According to Adamu et al. [87], crude extracts with IC_50_ ≥ 6 mg/mL are considered to have weak anthelmintic activities. Based on this assertion and categorization standard, it follows that the extracts in this study showed very high anthelmintic efficacy against *H. polygyrus* developmental life stages in vitro. Beyond the observed anthelmintic activity, the extracts exhibited considerable antioxidant potential, further supporting the pharmacological relevance of the phytochemical constituents identified in *T. superba*.

### 4.2. Antioxidant Activity

Biological systems maintain a delicate balance between oxidants and antioxidants to preserve cellular homeostasis, with free radicals playing a central role in this equilibrium [88, 89]. The potent antioxidant activity observed in the extracts of *T. superba* may be attributed to the hydroxyl groups of bioactive compounds such as tannins, phenols, and flavonoids, which neutralize free radicals through electron and hydrogen donation, thereby inhibiting lipid peroxidation and mitigating oxidative stress [32, 90]. In the present study, the antioxidant potential of the extracts was evaluated across four complementary assays, namely, DPPH, NO, H_2_O_2_ scavenging, and ferric reducing power, to comprehensively characterize their free radical neutralizing capacity.

The ethanolic extract demonstrated superior DPPH RSAactivity (IC_50_: 0.753 mg/mL) compared to both the aqueous extract (IC_50_: 6.263 mg/mL) and the reference standard vitamin C (IC_50_: 1.906 mg/mL), indicating that the ethanolic extract was the most potent radical scavenger among all tested samples. Higher DPPH RSA of the ethanolic extract could be due to higher content of flavonoids, phenolic compounds, and other antioxidant phytochemicals, which are extracted more efficiently in ethanol. This finding is consistent with the observation of Momo et al. [31], who similarly reported the hydroethanolic extract of *T. superba* as exhibiting the highest DPPH RSA among compared plant extracts. However, the present results contrast with those of Elvire et al. [91], who reported the DPPH RSA of *T. superba* bark extract to be lower than that of ascorbic acid, a discrepancy that may be attributed to the differences in extraction methods, plant part used, or geographical origin of the plant material. NO radical inhibition was also higher with the ethanolic extract (IC_50_: 3.8 μg/mL) than the aqueous extract (IC_50_: 245.5 μg/mL) and even much better than that of the standard antioxidant, ascorbic acid, although its IC_50_ value could not be determined. The enhanced NO scavenging activity in the ethanolic extract could be attributed to the higher extraction efficiency of bioactive phytochemicals such as flavonoids, phenolics, and other antioxidant compounds that are more soluble in ethanol than water. The good antioxidant activities in the DPPH and NO scavenging assays may be due to the high phenolic and flavonoid contents identified in both extracts. These compounds are capable of donating hydrogen atoms and electrons, thereby neutralizing free radicals via hydrogen atom transfer (HAT) and single electron transfer (SET) mechanisms [64, 92].

However, the ferric reducing power of the aqueous (IC_50_: 438.0 mg/mL) and ethanol extracts (IC_50_: 186.8 mg/mL) of *T*. *superba* was low compared to the standard antioxidant ascorbic acid (IC_50_: 35.19 mg/mL), with the activities of the extracts being dose dependent. These findings are similar to those observed by other authors [93, 94] but differ from that of Momo et al. [31] who had the highest FRAP activity with the aqueous extract of *T*. *superba* root extracts among other plant extracts. The lower reducing power of the aqueous extract (IC_50_: 438.0 μg/mL) compared to the ethanolic extract (IC_50_: 186.8 μg/mL) likely reflects the differential solubility of polyphenolic compounds in ethanol versus water. Ethanol preferentially extracts flavonoids, tannins, and phenolic acids with greater electron‐donating capacity, while aqueous extraction yields predominantly polar constituents such as glycosides and saponins with comparatively limited ferric reducing activity [95, 96]. The ethanolic extract had a high activity in DPPH assay, but a relatively low activity in reducing power assay. The difference could be attributed to the different mechanisms of the two assays. DPPH assay is mainly used to estimate the free radical scavenging ability of a sample by hydrogen atom or electron donation. The reducing power assay is used to estimate the reducing potential of compounds toward ferric ion. Hence, the RSA of some phytochemicals in ethanolic extract may be higher than their ferric reducing capacity, which may be the reason for the variation in IC_50_ values of the assays.

The hydrogen peroxide (H_2_O_2_) scavenging activity of *T. superba* extracts was very low compared to that of ascorbic acid, with the aqueous extract being more reactive than the ethanolic extract. The relatively higher H_2_O_2_ scavenging activity of the aqueous extract could be due to its higher total flavonoid contents in addition to its total phenolic content. The weaker H_2_O_2_ scavenging activity of the ethanolic extract (IC_50_: 8.672 μg/mL) relative to its superior DPPH potency (IC_50_: 0.753 μg/mL) reflects fundamental mechanistic differences between the two assays. This is because DPPH scavenging relies on rapid hydrogen atom and electron transfer to which flavonoids are particularly reactive, whereas H_2_O_2_ neutralization depends more on catalytic and nucleophilic properties of antioxidant compounds [14, 97, 98]. Interestingly, a slightly higher H_2_O_2_ scavenging activity was observed for the aqueous extract (6.949 μg/mL) than for the ethanolic extract (8.672 μg/mL), which was opposite to the trends in the DPPH and FRAP assays. The reason for this may be that polar phenolic compounds such as catechins, phenolic acids, and hydrophilic tannins are more soluble in water, and they can effectively neutralize H_2_O_2_ through nucleophilic and enzyme‐mimetic mechanisms. These mechanisms are different from those involved in DPPH radical quenching and ferric ion reduction [14, 43, 98].

The present findings therefore point out that the ethanolic extract showed higher DPPH and NO radical scavenging than the aqueous extract, whereas the aqueous extract had slightly higher H_2_O_2_ scavenging. Reducing power activity was moderate for both extracts compared to ascorbic acid. Furthermore, the ethanolic extract of *T. superba* displayed superior NO and DPPH scavenging compared to standard vitamin C, suggesting strong potential as a natural antioxidant source.

### 4.3. UHPLC‐MS/MS Profiling and *In Silico* Characterization of Bioactive Candidates

Molecular docking complemented the UHPLC‐MS/MS profiling by identifying phytochemicals with strong theoretical interactions toward *β*‐tubulin and SDH, two key parasitic targets. Among the 82 identified compounds, kaempferol, luteolin, termilignan, and brevifolin‐carboxylic acid exhibited the most favorable binding affinities, suggesting a possible contribution to the larvicidal activity observed experimentally, particularly against L2 and L3 larval stages. These interactions point to potential inhibition of parasite cytoskeletal integrity and energy metabolism, respectively. Overall, the correlation of docking scores with biological activity underscores the likely contribution of these phytochemicals to the anthelmintic activity of *T. superba* extracts.

Molecular docking revealed that the selected phytocompounds, kaempferol, brevifolin‐carboxylic acid, luteolin, scutellarein, and (−)‐epicatechin, interacted with *β*‐tubulin via hydrogen bonds with residues Asn101, Asn206, Asn228, Asp179, and Gln11, and through π–π interactions at Tyr224. Gln11 is a recognized binding site corroborated by previous studies [73, 99]. Since *β*‐tubulin is integral to microtubule integrity, governing parasite motility, cell division, and nutrient uptake, its disruption impairs polymerization, critical for parasite growth, which is the mechanism of benzimidazoles such as albendazole [100]. Notably, whereas albendazole (positive control) formed only a single hydrogen bond with Asp179, all five phytocompounds exhibited broader binding interactions, suggesting that these compounds may contribute to the anthelmintic activity by targeting *β*‐tubulin and disrupting essential parasite functions.

The molecular docking results of the present study show meaningful convergence for *β*‐tubulin with findings reported by Serena et al. [73]. Residues such as Asn228, Asn206, Gln11, Tyr224, and Asn101 were recurrently engaged across both studies by structurally diverse plant‐derived compounds, with Tyr224 consistently interacting through pi–pi stacking, a feature central to ligand stabilization within the colchicine binding site.

The top‐ranked compounds showed favorable in silico ADMET profiles, including good drug likeness, acceptable oral bioavailability, and low predicted toxicity risks further supporting their safety for therapeutic use. These results support the docking findings by indicating that the ligands possess not only strong target‐binding abilities but also promising pharmacokinetic and safety properties, crucial for early stage drug discovery.

### 4.4. Cytotoxicity and Safety

Beyond anthelmintic potency, the safety profile of *T. superba* extracts was evaluated to assess their cytotoxic potential against mammalian cells. The erythrocyte model is extensively used for evaluating cytotoxicity on red blood cells due to its ability to offer a rapid and comprehensive indication of membrane toxicity [101]. Many researchers have utilized this model to investigate the interactions between drugs, plant extracts, and membranes [102]. Both extracts showed relatively low hemolytic activities with percentage hemolysis less than 8%, with a concentration‐dependent increase. The aqueous extract was comparatively less cytotoxic with just 2% hemolysis against 7.6% for the ethanolic extract at the highest concentration of 1000 μg/mL. The low hemolytic activities of both extracts are proof of their safety. This slightly higher hemolytic activity of the ethanolic extract compared to the aqueous extract may probably be due to the preferential extraction of membrane‐disrupting phytochemicals by ethanol, especially tannins and saponins, which destabilize erythrocyte membranes by precipitating proteins and interacting with cholesterol, respectively [62, 103, 104]. The negligible increase in the hemolytic activity of the aqueous extract (1.8%–2.0%) is in line with the limited membrane‐disrupting ability of its predominantly hydrophilic constituents. Similar results were obtained by Andleeb et al. [105], who obtained minimal hemolytic effect of *Centratherum anthelminticum* seed extracts on human and chicken red blood cells.

## 5. Conclusion

This study validates the traditional use of these plant extracts by the local populations and native healers in the treatment of intestinal worm infections. The ethanolic and aqueous extracts of *T. superba* demonstrated measurable antioxidant and anthelmintic activities, supported by a rich phytochemical profile of flavonoids, phenols, tannins, alkaloids, and quinones. The ethanolic extract showed a superior RSA in the DPPH and NO assays, with the aqueous extract exhibiting greater ovicidal and L1 larvicidal activity. This study provides novel insights into the relative sensitivity of L2 larvae and the differential contribution of flavonoids and quinones to stage‐specific anthelmintic activity. Both extracts were confirmed safe below the 10% hemolysis threshold. Molecular docking further identified kaempferol, luteolin, and termilignan as promising lead anthelmintic candidates, recording superior binding affinities toward *β*‐tubulin and SDH compared to albendazole. This was supported by favorable ADME and safety profiles, collectively highlighting their potential as lead compounds for future anthelmintic drug development. These findings highlight *T*. *superba*’s potential as a promising anthelmintic agent. However, further investigations such as in vivo and acute toxicity studies are important to support these observations.

## Author Contributions

Cédric Yamssi, Nadia A. C. Noumedem, Joan Echi Ebanga Eyong, Vincent Khan Payne, and Helen Kuokuo Kimbi conceived the idea and designed the experiments. Bismarck Nganji Nsa, Yan Wu, Tientcheu N. J. Sandra, Gamago N. Guy‐Armand, Mounvera A. Azizi, and Ngouyamsa N. A. Sidiki performed the experiments. Bismarck Nganji Nsa, Joan Echi Ebanga Eyong, Haibo Hu, and Cédric Yamssi analyzed and interpreted the data. Nadia A. C. Noumedem, Cédric Yamssi, Bismarck Nganji Nsa Haibo Hu, and Vincent Khan Payne drafted the manuscript.

## Funding

The study received no funding.

## Disclosure

All authors read and approved the final manuscript.

## Conflicts of Interest

The authors declare no conflicts of interest.

## Practical Application

The accuracy of the IC_50_ estimates is diminished for certain samples, especially when values are outside the tested concentration range. This limitation has been expressly recognized to prevent overinterpretation of the findings.

## Data Availability

Data are available on request from the authors.

## References

[1] Else K. J. , Keiser J. , and Holland C. V. , Whipworm and Roundworm Infections, Nature Reviews Disease Primers. (2020) 6, no. 1, 10.1038/s41572-020-0171-3. 32467581

[2] Aula O. P. , McManus D. P. , Jones M. K. , and Gordon C. A. , Schistosomiasis with a Focus on Africa, Tropical medicine and infectious disease. (2021) 6, no. 3, 10.3390/tropicalmed6030109. PMC8293433 34206495

[3] WHO , Soil-Transmitted Helminth Infections, 2023, https://www.who.int/news-room/fact-sheets/detail/soil-transmitted-helminth-infections.

[4] Bismarck N. , Payne V. , Cedric Y. , and Nadia N. , Gastro-Intestinal Helminth Infections and Associated Risk Factors Amongst School Aged Children in Kouoptamo Noun Division, West Region, Cameroon, Int. Arch. Public Health Community Med. (2020) 4.

[5] Humphries D. , Nguyen S. , Boakye D. , Wilson M. , and Cappello M. , The Promise and Pitfalls of Mass Drug Administration to Control Intestinal Helminth Infections, Current Opinion in Infectious Diseases. (2012) 25, no. 5, 584–589, 10.1097/qco.0b013e328357e4cf. 22903231

[6] Reyes-Guerrero D. E. , Olmedo-Juárez A. , and Mendoza-de Gives P. , Control and Prevention of Nematodiasis in Small Ruminants: Background, Challenges and Outlook in Mexico, Revista mexicana de ciencias pecuarias. (2021) 12, 186–204.

[7] Olsen A. , van Lieshout L. , and Marti H. , Strongyloidiasis–The Most Neglected of the Neglected Tropical Diseases?, Transactions of the Royal Society of Tropical Medicine and Hygiene. (2009) 103, no. 10, 967–972, 10.1016/j.trstmh.2009.02.013. 19328508

[8] Geary T. G. , Are New Anthelmintics Needed to Eliminate Human Helminthiases?, Current Opinion in Infectious Diseases. (2012) 25, no. 6, 709–717, 10.1097/qco.0b013e328359f04a. 23041774

[9] Williams A. R. , Soelberg J. , and Jäger A. K. , Anthelmintic Properties of Traditional African and Caribbean Medicinal Plants: Identification of Extracts with Potent Activity Against *Ascaris suum* in Vitro, Parasite. (2016) 23, 10.1051/parasite/2016024. PMC4908306 27301442

[10] Manolaraki F. , Propriétés Anthelminthiques Du Sainfoin (Onobrychis Viciifoliae): Analyse Des Facteurs De Variations Et Du Rôle Des Composés Phénoliques Impliqués, 2011, Institut National Polytechnique de Toulouse-INPT.

[11] Sadiq I. Z. , Free Radicals and Oxidative Stress: Signaling Mechanisms, Redox Basis for Human Diseases, and Cell Cycle Regulation, Current Molecular Medicine. (2023) 23, no. 1, 13–35, 10.2174/1566524022666211222161637. 34951363

[12] Yehia S. A. , Badr A. M. , Bashtar A.-R. , Ibrahim M. A.-A. , Mousa M. R. , and Mostafa N. A. , Immune Response, Oxidative Stress, and Histological Changes of Wistar Rats After Being Administered with Parascaris equorum Antigen, Scientific Reports. (2024) 14, no. 1, 10.1038/s41598-024-67788-y. PMC11300452 39103392

[13] Chandimali N. , Bak S. G. , and Park E. H. , Free Radicals and Their Impact on Health and Antioxidant Defenses: a Review, Cell Death Discovery. (2025) 11, no. 1, 10.1038/s41420-024-02278-8. PMC11760946 39856066

[14] Halliwell B. and Gutteridge J. M. , Free Radicals in Biology and Medicine, 2015, Oxford University Press.

[15] Hoste H. , Jackson F. , Athanasiadou S. , Thamsborg S. M. , and Hoskin S. O. , The Effects of tannin-rich Plants on Parasitic Nematodes in Ruminants, Trends in Parasitology. (2006) 22, no. 6, 253–261, 10.1016/j.pt.2006.04.004. 16632404

[16] Megnigueu E. M. , Nyemb N. J. , and Ngwasiri N. N. , In Vitro Anthelmintic Activities of Extracts and Fractions of Cosmos sulphureus Cav, Against Onchocerca ochengi, Journal of Diseases and Medicinal Plants. (2020) 6, no. 1, 22–30.

[17] Romero-Benavides J. C. , Ruano A. L. , Silva-Rivas R. , Castillo-Veintimilla P. , Vivanco-Jaramillo S. , and Bailon-Moscoso N. , Medicinal Plants Used as Anthelmintics: Ethnomedical, Pharmacological, and Phytochemical Studies, European Journal of Medicinal Chemistry. (2017) 129, 209–217, 10.1016/j.ejmech.2017.02.005. 28231520

[18] Tabi M. M. , Powell M. , and Hodnicki D. , Use of Traditional Healers and Modern Medicine in Ghana, International Nursing Review. (2006) 53, no. 1, 52–58, 10.1111/j.1466-7657.2006.00444.x. 16430761

[19] WHO , WHO Global Report on Traditional and Complementary Medicine 2019, 2019, World Health Organization.

[20] Mali R. G. and Mehta A. A. , A Review on Anthelmintic Plants, 2008.

[21] Groulez J. and Wood P. J. , Terminalia Superba: A Monograph, 1985.

[22] Adjanohoun J. , Aboubakar N. , and Dramane K. , Traditional Medicine and Pharmacopoeia: Contribution to Ethnopharmacological and Floristic Studies in Cameroon, 1996, OAU/STRC.

[23] Wansi J. D. , Lallemand M.-C. , and Chiozem D. D. , α-Glucosidase Inhibitory Constituents from Stem Bark of Terminalia superba (Combretaceae), Phytochemistry. (2007) 68, no. 15, 2096–2100, 10.1016/j.phytochem.2007.02.020. 17434189

[24] Anago E. , Activitésantibactériennes De Quelquesplantes De La Pharmacopéeafricainesur Des Souches De E. coli Productrices De Bêtalactamases Thèse de Doctorat en Biochimie à l’Universitéd’Abomey-calavi, 2009.

[25] Kuete V. , Tabopda T. K. , Ngameni B. , Nana F. , Tshikalange T. E. , and Ngadjui B. T. , Antimycobacterial, Antibacterial and Antifungal Activities of Terminalia superba (Combretaceae), South African Journal of Botany. (2010) 76, no. 1, 125–131, 10.1016/j.sajb.2009.09.009.

[26] Ahon M. G. , Akapo-Akue J. M. , Kra M. A. , Ackah J. B. , Zirihi N. G. , and Djaman J. A. , Antifungal Activity of the Aqueous and hydro-alcoholic Extracts of Terminalia superba Engl. on the in Vitro Growth of Clinical Isolates of Pathogenic Fungi, Agriculture and Biology Journal of North America. (2011) 2, no. 2, 250–257.

[27] Dongmo A. , Beppe J. , Nole T. , and Kamanyi A. , Analgesic Activities of the Stem Bark Extract of Terminalia Superba Engl. Et Diels (Combretaceae), Pharmacologyonline. (2006) 2, 171–177.

[28] Sarwar S. , Khatun A. , Nahar K. , and Rahman M. A. , Phytochemical Screening, Cytotoxic and Anthelmintic Activities of the Methanolic Leaf Extract of *Terminalia catappa* Linn, Journal of Pharmacognosy and Phytochemistry. (2016) 5, no. 6, 24–27.

[29] Busari I. , Soetan K. , Aiyelaagbe O. , and Babayemi O. , Phytochemical Screening and in Vitro Anthelmintic Activity of Methanolic Extract of Terminalia glaucescens Leaf on *Haemonchus contortus* Eggs, Acta Tropica. (2021) 223, 10.1016/j.actatropica.2021.106091. 34389333

[30] Cédric Y. , Yaghoobi M. , and Nadia N. A. C. , In Vitro Anthelmintic Activity of Ethanol and Aqueous Extracts of Terminalia macroptera and Bridelia micrantha Against Heligmosomoides polygyrus, *Caenorhabditis elegans* and in-silico Molecular Docking Evaluation of Some Isolated Phytoconstituents, South African Journal of Botany. (2024) 164, 356–365, 10.1016/j.sajb.2023.11.053.

[31] Momo C. E. N. , Ngwa A. F. , Dongmo G. I. F. , and Oben J. E. , Antioxidant Properties and α-amylase Inhibition of Terminalia superba, Albizia Sp., Cola nitida, Cola odorata and Harungana madagascarensis Used in the Management of Diabetes in Cameroon, Journal of Health Science. (2009) 55, no. 5, 732–738, 10.1248/jhs.55.732.

[32] Ani N. I. , Okolo K. O. , and Offiah R. O. , Evaluation of Antibacterial, Antioxidant, and Anti-inflammatory Properties of GC/MS Characterized Methanol Leaf Extract of Terminalia superba (Combretaceae, Engl. & Diels), Future Journal of Pharmaceutical Sciences. (2023) 9, no. 1.

[33] Behnke J. M. , Menge D. , and Noyes H. , Heligmosomoides Bakeri: a Model for Exploring the Biology and Genetics of Resistance to Chronic Gastrointestinal Nematode Infections, Parasitology. (2009) 136, no. 12, 1565–1580, 10.1017/s0031182009006003. 19450375

[34] Reynolds L. A. , Filbey K. J. , and Maizels R. M. , Immunity to the Model Intestinal Helminth Parasite Heligmosomoides polygyrus, Seminars in Immunopathology. (2012) . 10.1007/s00281-012-0347-3 PMC3496515 23053394

[35] Wabo-Poné J. , Yondo J. , Fossi T. , Komtangi M. C. , Bilong-Bilong C. , and Mpoame M. , The in Vitro Effects of Chenopodium ambrosioides (Chenopodiaceae) Extracts on the Parasitic Nematode Heligmosomoides bakeri (Nematoda, Heligmosomatidae), Journal of Pharmacognosy and Phytotherapy. (2011) 3, no. 4, 56–62.

[36] Cédric Y. , Nadia N. A. C. , Nfufu S. , Azizi M. A. , Sandra T. N. J. , and Payne V. K. , Nematocidal Activity of Ethanol and Aqueous Extracts of Persea americana Seeds Against Heligmosomoides polygyrus Using the Worm Microtracker Method, Journal of Parasitology Research. (2023) 2023, no. 1, 9545565–6, 10.1155/2023/9545565. PMC10513806 37745983

[37] Johnston C. J. , Robertson E. , and Harcus Y. , Cultivation of Heligmosomoides Polygyrus: an Immunomodulatory Nematode Parasite and Its Secreted Products, Journal of Visualized Experiments. (2015) no. 98, 10.3791/52412-v. PMC4401400 25867600

[38] Ahmed A. , Al-Mekhlafi H. M. , Al-Adhroey A. H. , Ithoi I. , Abdulsalam A. M. , and Surin J. , The Nutritional Impacts of soil-transmitted Helminths Infections Among Orang Asli Schoolchildren in Rural Malaysia, Parasites & Vectors. (2012) 5, no. 1, 10.1186/1756-3305-5-119. PMC3419660 22704549

[39] Popovici C. , Saykova I. , and Tylkowski B. , Evaluation De L’Activité Antioxydant Des Composés Phénoliques Par La Réactivité Avec Le Radical Libre DPPH, Revue de génie industriel. (2009) 4, no. 8, 25–39.

[40] Bokhari J. , Khan M. R. , Shabbir M. , Rashid U. , Jan S. , and Zai J. A. , Evaluation of Diverse Antioxidant Activities of Galium aparine, Spectrochimica Acta Part A: Molecular and Biomolecular Spectroscopy. (2013) 102, 24–29, 10.1016/j.saa.2012.09.056. 23211618

[41] Ashokkumar D. , Thamilselvan V. , Gp S. , Mazumder U. K. , and Gupta M. , Antioxidant and Free Radical Scavenging Effects of Lippia nodiflora, Pharmaceutical Biology. (2008) 46, no. 10-11, 762–771, 10.1080/13880200802315444.

[42] Suluvoy J. K. and Berlin G. V. , Phytochemical Profile and Free Radical Nitric Oxide (NO) Scavenging Activity of Averrhoa bilimbi L. Fruit Extract, 3 Biotech. (2017) 7, no. 1, 10.1007/s13205-017-0678-9. PMC5429310 28500407

[43] Ruch R. J. , Cheng S.-j. , and Klaunig J. E. , Prevention of Cytotoxicity and Inhibition of Intercellular Communication by Antioxidant Catechins Isolated from Chinese Green Tea, Carcinogenesis. (1989) 10, no. 6, 1003–1008, 10.1093/carcin/10.6.1003. 2470525

[44] Haddouchi F. , Chaouche T. , and Halla N. , Phytochemical Screening, Antioxidant Activities and Hemolytic Power of Four Saharan Plants from Algeria, Phytothérapie. (2018) 16, no. 1, 254–262.

[45] Shaikh J. R. and Patil M. , Qualitative Tests for Preliminary Phytochemical Screening: an Overview, International Journal of Chemical Studies. (2020) 8, no. 2, 603–608, 10.22271/chemi.2020.v8.i2i.8834.

[46] Mbaebie B. , Edeoga H. , and Afolayan A. , Phytochemical Analysis and Antioxidants Activities of Aqueous Stem Bark Extract of Schotia latifolia Jacq, Asian Pacific Journal of Tropical Biomedicine. (2012) 2, no. 2, 118–124, 10.1016/s2221-1691(11)60204-9. PMC3609248 23569880

[47] Zhishen J. , Mengcheng T. , and Jianming W. , The Determination of Flavonoid Contents in Mulberry and Their Scavenging Effects on Superoxide Radicals, Food Chemistry. (1999) 64, no. 4, 555–559, 10.1016/s0308-8146(98)00102-2.

[48] Abongwa M. , Martin R. J. , and Robertson A. P. , A Brief Review on the Mode of Action of Antinematodal Drugs, Acta Veterinaria. (2017) 67, no. 2, 137–152, 10.1515/acve-2017-0013. PMC5798647 29416226

[49] Khairuzzaman M. , Hasan M. M. , and Ali M. T. , Anthelmintic Screening of Bangladeshi Medicinal Plants and Related Phytochemicals Using in Vitro and in Silico Methods: an Ethnobotanical Perspective, Journal of Ethnopharmacology. (2024) 328, 10.1016/j.jep.2024.118132. 38565411

[50] Cheng W. , Yan Y. , and Xiao T. , Design, Synthesis and Inhibitory Activity of Novel 2, 3-dihydroquinolin-4 (1H)-one Derivatives as Potential Succinate Dehydrogenase Inhibitors, European Journal of Medicinal Chemistry. (2021) 214, 10.1016/j.ejmech.2021.113246. 33582385

[51] Wang J. , Lu T. , and Xiao T. , Novel quinolin‐2 (1 H)‐One Analogues as Potential Fungicides Targeting Succinate Dehydrogenase: Design, Synthesis, Inhibitory Evaluation and Molecular Modeling, Pest Management Science. (2023) 79, no. 10, 3425–3438, 10.1002/ps.7332. 36562216

[52] Liu W. , Shao H. , and Qi D. , The New Nematicide Cyclobutrifluram Targets the Mitochondrial Succinate Dehydrogenase Complex in Bursaphelenchus Xylophilus, International Journal of Molecular Sciences. (2024) 25, no. 13, 10.3390/ijms25136914. PMC11241274 39000026

[53] Olsson M. H. , Søndergaard C. R. , Rostkowski M. , and Jensen J. H. , PROPKA3: Consistent Treatment of Internal and Surface Residues in Empirical P K a Predictions, Journal of Chemical Theory and Computation. (2011) 7, no. 2, 525–537, 10.1021/ct100578z. 26596171

[54] Schrödinger M. , Schrödinger: Version 11.0, 2015, Schrödinger, LLC, New York, NY, USA.

[55] Shelley J. C. , Cholleti A. , Frye L. L. , Greenwood J. R. , Timlin M. R. , and Uchimaya M. , Epik: a Software Program for Pk a Prediction and Protonation State Generation for Drug-like Molecules, Journal of Computer-Aided Molecular Design. (2007) 21, no. 12, 681–691, 10.1007/s10822-007-9133-z. 17899391

[56] Friesner R. A. , Banks J. L. , and Murphy R. B. , Glide: a New Approach for Rapid, Accurate Docking and Scoring. 1. Method and Assessment of Docking Accuracy, Journal of Medicinal Chemistry. (2004) 47, no. 7, 1739–1749, 10.1021/jm0306430. 15027865

[57] Chikhale H. U. and Rishipathak D. D. , In Silico Prediction, Molecular Docking Study for Identification of Novel Nitrogen Substituted Benzoxazole Derivative for Their Potential Biological Activity, Chemistry Africa. (2025) 8, no. 3, 1–13, 10.1007/s42250-025-01208-0.

[58] Dash R. , Hosen S. Z. , and Karim M. R. , In Silico Analysis of indole-3-carbinol and its Metabolite DIM as EGFR Tyrosine Kinase Inhibitors in Platinum Resistant Ovarian Cancer Vis a Vis ADME/T Property Analysis, Journal of Applied Pharmaceutical Science. (2015) 5, no. 11, 073–078, 10.7324/japs.2015.501112.

[59] Tiwari A. , Phytochemical Profiling and Anthelmintic Activity of Manilkara zapota L.(Chiku) Extracts: An Integrated In-vitro and In-silico Approaches, 2024.

[60] Zhang L. , Yuan B. , Wang H. , and Gao Y. , Therapeutic Effect of Agaricus brasiliensis on Phenylhydrazine‐Induced Neonatal Jaundice in Rats, Biomed Research International. (2015) 2015, no. 1, 651218–6, 10.1155/2015/651218. PMC4389989 25883968

[61] Tamo S. , Essama R. , and Etoa F. , Plants Used in Bandjoun Village (La’Djo) to Cure Infectious Diseases: an Ethnopharmacology Survey and in-vitro Time-Kill Assessment of Some of Them Against *Escherichia coli* , The J Phytopharmacol. (2016) 5, no. 2, 56–70, 10.31254/phyto.2016.5205.

[62] Chung K.-T. , Wong T. Y. , Wei C.-I. , Huang Y.-W. , and Lin Y. , Tannins and Human Health: a Review, Critical Reviews in Food Science and Nutrition. (1998) 38, no. 6, 421–464, 10.1080/10408699891274273. 9759559

[63] Hoste H. , Martinez-Ortiz-De-Montellano C. , and Manolaraki F. , Direct and Indirect Effects of Bioactive tannin-rich Tropical and Temperate Legumes Against Nematode Infections, Veterinary Parasitology. (2012) 186, no. 1-2, 18–27, 10.1016/j.vetpar.2011.11.042. 22188981

[64] Pisoschi A. M. and Pop A. , The Role of Antioxidants in the Chemistry of Oxidative Stress: a Review, European Journal of Medicinal Chemistry. (2015) 97, 55–74, 10.1016/j.ejmech.2015.04.040. 25942353

[65] Evans W. C. , Trease and Evans’ Pharmacognosy, 2009, Elsevier Health Sciences.

[66] Tiwari P. , Kumar B. , Kaur M. , Kaur G. , and Kaur H. , Phytochemical Screening and Extraction: a Review, Internationale Pharmaceutica Sciencia. (2011) 1, no. 1, 98–106.

[67] Cowan M. M. , Plant Products as Antimicrobial Agents, Clinical Microbiology Reviews. (1999) 12, no. 4, 564–582, 10.1128/cmr.12.4.564. PMC88925 10515903

[68] Katiki L. M. , Ferreira J. F. , and Gonzalez J. M. , Anthelmintic Effect of Plant Extracts Containing Condensed and Hydrolyzable Tannins on *Caenorhabditis elegans*, and Their Antioxidant Capacity, Veterinary Parasitology. (2013) 192, no. 1-3, 218–227, 10.1016/j.vetpar.2012.09.030. 23102761

[69] Enejoh O. S. , Shuaibu K. , Suleiman M. , and Ajanusi J. , Evaluation of Anthelmintic Efficacy of Citrus Aurantifolia (Christm) Fruit Juice Against Heligmosomoides bakeri, International of Advanced Biological Research. (2014) 4, no. 4, 448–445.

[70] Santa R. S. , Santos F. , and Lima H. , In Vitro Anthelmintic and Cytotoxic Activities of Extracts of Persea willdenovii Kosterm (Lauraceae), Journal of Helminthology. (2018) 92, no. 6, 674–680. 10.1017/S0022149X17000979 29067895

[71] Baigomen C. , Besati M. , and Christelle Nadia N. A. , In Vitro Anthelmintic Activities of Khaya anthotheca and Faidherbia albida Extracts Used in Chad by Traditional Healers for the Treatment of Helminthiasis and in Silico Study of Phytoconstituents, Journal of Tropical Medicine. (2024) 2024, no. 1. 10.1155/2024/8564163 PMC11226339 38974476

[72] Noumedem C. N. , Yaghoobi M. , and Cédric Y. , Anthelmintic Activity of Ethanolic and Aqueous Extracts of Khaya grandifoliola Stem Bark Against Heligmosomoides Polygyrus: In Vitro and in Silico Approaches, 2024. 10.1155/2024/6735764 PMC11268970 39050406

[73] Serena N. N. , Besati M. , and Nadia N. A. C. , In Vitro and in Silico Anthelmintic Activity of Extracts of Lannea kerstingii and Ficus thonningii on Heligmosomoides polygyrus, Journal of Parasitology Research. (2024) 2024, no. 1, 10.1155/2024/1858154. PMC11316912 39131749

[74] Carvalho C. O. , Chagas A. C. S. , and Cotinguiba F. , The Anthelmintic Effect of Plant Extracts on *Haemonchus contortus* and Strongyloides venezuelensis, Veterinary Parasitology. (2012) 183, no. 3-4, 260–268, 10.1016/j.vetpar.2011.07.051. 21872995

[75] Kollins E. , Wabo P. , Payne V. , Jeannette Y. , and Marie-Claire K. , In Vitro Comparative Effect of Aqueous (Cold and Hot Water) and Ethanolic Extracts of the Seeds of Aframomum Danielli (Zingiberaceae) on Three Life Cycle Stages of the Parasitic Nematode Heligmosomoides bakeri (Nematoda; Heligmosomatidae), Parasite of the Laboratory Mice (Mus Musculus), Med Arm Pl. (2012) 1, no. 111, 2167–0412.1000111.

[76] Jimenez-Cisneros B. and Maya-Rendon C. , Helminths and Sanitation, Communicating current research and educational topics and trends in applied microbiology. (2007) 1, 60–71.

[77] Komtangi M. , Yondo J. , Poné J. W. , Tane P. , and Mbida M. , In Vitro Anthelmintic Activity of Vepris louisii Gilbert Extracts on Developmental Stages of Heligmosomoides bakeri (Nematoda: Heligmosomoidae), Medicinal Plants-International Journal of Phytomedicines and Related Industries. (2014) 6, no. 3, 174–180, 10.5958/0975-6892.2014.00005.7.

[78] Athanasiadou S. , Kyriazakis I. , Jackson F. , and Coop R. , Direct Anthelmintic Effects of Condensed Tannins Towards Different Gastrointestinal Nematodes of Sheep: in Vitro and in Vivo Studies, Veterinary Parasitology. (2001) 99, no. 3, 205–219, 10.1016/s0304-4017(01)00467-8. 11502368

[79] Wabo P. J. , Payne V. , and Mbogning T. G. , In Vitro Anthelminthic Efficacy of Dichrocephala integrifolia (Asteraceae) Extracts on the gastro-intestinal Nematode Parasite of Mice: Heligmosomoides bakeri (Nematoda, Heligmosomatidae), Asian Pacific Journal of Tropical Biomedicine. (2013) 3, no. 2, 100–104, 10.1016/s2221-1691(13)60032-5. PMC3627168 23593587

[80] Poné J. W. , Bilong C. B. , and Mpoame M. , In Vitro Nematicidal Activity of Extracts of Canthium mannii (Rubiaceae), on Different life-cycle Stages of Heligmosomoides polygyrus (Nematoda, Heligmosomatidae), Journal of Helminthology. (2010) 84, no. 2, 156–165, 10.1017/s0022149x09990435. 19728896

[81] Ngouateu T. S. E. , Nmbogning Tayo G. , and Ngangout Alidou M. , Anthelminthic Properties of Methylene chloride-methanol (1: 1) Extracts of Two Cameroonians Medicinal Plants on Heligmosomoides bakeri (Nematoda: Heligmosomatidea), BMC Complementary and Alternative Medicine. (2017) 17, no. 1, 10.1186/s12906-017-1908-8. PMC5553925 28800757

[82] Soulsby E. , Helminths, Arthropods and Protozoa III M of Domesticated Animals, Tindall. (1982) .

[83] Cushnie T. T. and Lamb A. J. , Antimicrobial Activity of Flavonoids, International Journal of Antimicrobial Agents. (2005) 26, no. 5, 343–356, 10.1016/j.ijantimicag.2005.09.002. PMC7127073 16323269

[84] Mutha R. E. , Tatiya A. U. , and Surana S. J. , Flavonoids as Natural Phenolic Compounds and Their Role in Therapeutics: an Overview, Future Journal of Pharmaceutical Sciences. (2021) 7, no. 1, 10.1186/s43094-020-00161-8. PMC7816146 33495733

[85] Hoste H. , Meza-Ocampos G. , and Marchand S. , Use of agro-industrial by-products Containing Tannins for the Integrated Control of Gastrointestinal Nematodes in Ruminants, Parasite. (2022) 29, 10.1051/parasite/2022010. PMC8884022 35225785

[86] Dias M. C. , Pinto D. C. , and Silva A. M. , Plant Flavonoids: Chemical Characteristics and Biological Activity, Molecules. (2021) 26, no. 17, 10.3390/molecules26175377. PMC8434187 34500810

[87] Adamu M. , Naidoo V. , and Eloff J. N. , Efficacy and Toxicity of Thirteen Plant Leaf Acetone Extracts Used in Ethnoveterinary Medicine in South Africa on Egg Hatching and Larval Development of *Haemonchus contortus* , BMC Veterinary Research. (2013) 9, no. 1, 10.1186/1746-6148-9-38. PMC3599279 23442744

[88] Metcalfe N. B. and Alonso‐Alvarez C. , Oxidative Stress as a Life‐History Constraint: the Role of Reactive Oxygen Species in Shaping Phenotypes from Conception to Death, Functional Ecology. (2010) 24, no. 5, 984–996, 10.1111/j.1365-2435.2010.01750.x.

[89] Sundaram Sanjay S. and Shukla A. K. , Free Radicals Versus Antioxidants, Potential Therapeutic Applications of nano-antioxidants, 2021, Springer, 1–17.

[90] Gülçın I. , Beydemır Ş. , Alici H. A. , Elmastaş M. , and Büyükokuroğlu M. E. , In Vitro Antioxidant Properties of Morphine, Pharmacological Research. (2004) 49, no. 1, 59–66. 10.1016/j.phrs.2003.07.012 14597153

[91] Elvire K. F. E. , Casimir A. D. , Durand D.-N. , Bawa B. , Lamine B. M. , and Frédéric L. , Antioxidant and Antibacterial Activities of Terminalia superba Engl. and Diels (Combretaceae) Bark Extracts, International Journal of Current Microbiology and Applied Sciences. (2018) 7, 2836–2846.

[92] Shahidi F. and Ambigaipalan P. , Phenolics and Polyphenolics in Foods, Beverages and Spices: Antioxidant Activity and Health effects–A Review, Journal of Functional Foods. (2015) 18, 820–897, 10.1016/j.jff.2015.06.018.

[93] Ismaili R. , Houbairi S. , Sanâa L. , Khadija M. , and Abdeslam L. , Etude De L’Activité Antioxydante Des Huiles Essentielles De Plantes Aromatiques Et Médicinales Marocaines, European Scientific Journal, ESJ. (2017) 13, no. 12, 10.19044/esj.2017.v13n12p323.

[94] Azizi A. M. , Christelle Nadia N. A. , and Cedric Y. , In Vitro Antiplasmodial, Cytotoxicity, and Antioxidant Activities of Lophira lanceolata (Ochnaceae): a Cameroonian Plant Commonly Used to Treat Malaria, Journal of Tropical Medicine. (2023) 2023, no. 1, 10.1155/2023/4061592. PMC9938790 36820149

[95] Surveswaran S. , Cai Y.-Z. , Corke H. , and Sun M. , Systematic Evaluation of Natural Phenolic Antioxidants from 133 Indian Medicinal Plants, Food Chemistry. (2007) 102, no. 3, 938–953, 10.1016/j.foodchem.2006.06.033.

[96] Kumar M. K. , Kaur G. , and Kaur H. , Internationale Pharmaceutica Sciencia, 2011.

[97] Huang D. , Ou B. , and Prior R. L. , The Chemistry Behind Antioxidant Capacity Assays, Journal of Agricultural and Food Chemistry. (2005) 53, no. 6, 1841–1856, 10.1021/jf030723c. 15769103

[98] Prior R. L. , Wu X. , and Schaich K. , Standardized Methods for the Determination of Antioxidant Capacity and Phenolics in Foods and Dietary Supplements, Journal of Agricultural and Food Chemistry. (2005) 53, no. 10, 4290–4302, 10.1021/jf0502698. 15884874

[99] Sharma O. P. , Pan A. , Hoti S. , Jadhav A. , Kannan M. , and Mathur P. P. , Modeling, Docking, Simulation, and Inhibitory Activity of the Benzimidazole Analogue Against β-tubulin Protein from *Brugia malayi* for Treating Lymphatic Filariasis, Medicinal Chemistry Research. (2012) 21, no. 9, 2415–2427, 10.1007/s00044-011-9763-5.

[100] Lacey E. , Mode of Action of Benzimidazoles, Parasitology Today. (1990) 6, no. 4, 112–115, 10.1016/0169-4758(90)90227-u. 15463312

[101] Podsiedlik M. , Markowicz-Piasecka M. , and Sikora J. , Erythrocytes as Model Cells for Biocompatibility Assessment, Cytotoxicity Screening of Xenobiotics and Drug Delivery, Chemico-Biological Interactions. (2020) 332, 10.1016/j.cbi.2020.109305. 33130048

[102] Jang J. H. , Harada H. , and Shibayama H. , A Randomized Controlled Trial Comparing Darbepoetin Alfa Doses in Red Blood Cell Transfusion-dependent Patients with low-or intermediate-1 Risk Myelodysplastic Syndromes, International Journal of Hematology. (2015) 102, no. 4, 401–412, 10.1007/s12185-015-1862-5. 26323997

[103] Shi J. , Arunasalam K. , Yeung D. , Kakuda Y. , Mittal G. , and Jiang Y. , Saponins from Edible Legumes: Chemistry, Processing, and Health Benefits, Journal of Medicinal Food. (2004) 7, no. 1, 67–78, 10.1089/109662004322984734. 15117556

[104] Smeriglio A. , Barreca D. , Bellocco E. , and Trombetta D. , Proanthocyanidins and Hydrolysable Tannins: Occurrence, Dietary Intake and Pharmacological Effects, British Journal of Pharmacology. (2017) 174, no. 11, 1244–1262, 10.1111/bph.13630. PMC5429339 27646690

[105] Andleeb R. , Ashraf A. , and Ijaz M. U. , In Vitro Antioxidant, Hemolytic, Thrombolytic Potencies of Centratherum anthelminticum Seed Extractsand It’s in Ovo Antiviral Efficacy, Pakistan Journal of Agricultural Sciences. (2020) 57, no. 5, 1261–1269.

